# Advances in FGF/FGFR Signaling: Implications for Disease and Therapy

**DOI:** 10.1002/mco2.70922

**Published:** 2026-08-22

**Authors:** Miaoyu Song, Xi Liu, Yang Xiao, Yongsheng Li, Huakan Zhao

**Affiliations:** ^1^ School of Medicine Chongqing University Cancer Hospital Chongqing University Chongqing China; ^2^ Department of Medical Oncology Chongqing University Cancer Hospital Chongqing China; ^3^ Key Laboratory of Chongqing Education Commission of China for Cancer Immunometabolism Translational Research Chongqing China; ^4^ Chongqing Key Laboratory for the Mechanism and Intervention of Cancer Metastasis Chongqing China; ^5^ Intelligent Oncology Innovation Center Designated by the Ministry of Education Chongqing University Chongqing China

**Keywords:** diseases, downstream signaling cascades, FGF/FGFR signaling, physiological functions, targeted intervention

## Abstract

The fibroblast growth factor (FGF)/FGF receptor (FGFR) signaling, primarily comprising FGFs, FGFRs, and the co‐receptor Klotho, regulates key physiological processes, including embryogenesis, tissue homeostasis, metabolism, and neurodevelopment. Dysregulation of FGF/FGFR signaling, whether resulting from pathological hyperactivation or loss‐of‐function, is implicated in multiple diseases such as skeletal dysplasia, metabolic disorders, cancers, and neurological disorders. Consequently, correction of causative FGF/FGFR signaling represents a promising clinical strategy. Despite the deepening understanding of the FGF/FGFR signaling, its clinical translation still faces challenges, including side effects and acquired resistance to pathway blockade, as well as safety concerns regarding FGF analogs. Herein, we provide an overview of FGF/FGFR signaling and summarize its cellular and physiological functions. We further delve into the relationship between aberrant (including hyperactivated or inactivated) FGF/FGFR signaling and diverse diseases. Additionally, we also discuss the strategies targeting the causative FGF/FGFR signaling, as well as offer new perspectives for optimizing these targeted therapies. Collectively, this review will contribute to the development of more precise strategies for the targeted regulation of dysregulated FGF/FGFR signaling.

## Graphical abstract

The FGF/FGFR signaling is indispensable for the maintenance of physiological homeostasis and governs multiple biological processes, including embryonic development, bone metabolism, angiogenesis, and neurogenesis, whereas aberrant hyperactivation of this pathway drives the progression of malignancies and autoimmune disorders, including inflammatory bowel disease (IBD). However, insufficient FGF/FGFR signaling is closely linked to metabolic and neurodegenerative diseases, encompassing metabolic dysfunction‐associated steatohepatitis (MASH) and Parkinson's disease (PD). Targeted therapeutic strategies, including FGFR inhibitors, monoclonal antibodies, CAR‐T‐cell therapy, FGF analogs, aptamers, and gene delivery approaches, are dedicated to correct the dysregulated FGF/FGFR signaling under distinct pathological conditions.

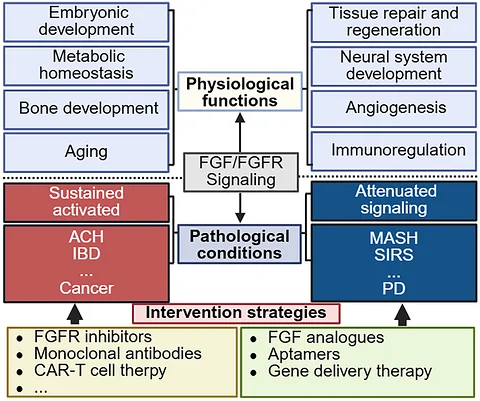

## 1 Introduction

Fibroblast growth factor (FGF) signaling is initiated primarily through the binding of FGF ligand to FGF receptor (FGFR), a family of transmembrane tyrosine kinases, which often act in concert with co‐receptors such as Klotho proteins [1, 2]. This interaction triggers multiple downstream signaling cascades, including the rat sarcoma virus/mitogen‐activated protein kinase (RAS/MAPK), phosphatidylinositol 3‐kinase/AKT (PI3K/AKT), phospholipase C gamma (PLCγ), and Janus kinase/signal transducer and activator of transcription (JAK/STAT) pathways. Through these tightly coordinated and often cross‐regulated networks, the FGF/FGFR signaling governs fundamental cellular functions, including proliferation, differentiation, migration, survival, and metabolism [3]. Moreover, it plays an indispensable role in embryonic morphogenesis, systemic metabolic homeostasis, postnatal tissue homeostasis, and injury‐induced regenerative responses [4]. Nevertheless, dysregulation of FGF/FGFR signaling, whether caused by its loss‐of‐function or pathological hyperactivation, disrupts physiological homeostasis and is implicated in diverse pathologies, including metabolic syndromes, skeletal dysplasia, malignancies, and neurodegenerative diseases [5]. For instance, impaired FGF19/FGFR4 signaling fails to suppress hepatic bile acid (BA) synthesis, leading to intrahepatic cholestasis [6]. Conversely, oncogenic FGFR2 mutations constitutively activate downstream effectors, such as Ras–Raf–MEK–ERK, PI3K–AKT, and JAK–STAT pathways, thereby driving cholangiocarcinoma initiation and progression [7].

Currently, targeted correction of the dysregulated FGF/FGFR signaling has emerged as one of the pivotal focuses in clinical translational research [8]. Although FGF/FGFR signaling‐related intervention strategies have demonstrated breakthrough efficacy in several clinical trials, their safety and effectiveness remain challenging. For instance, the engineered FGF19 analog NGM282 significantly ameliorates hepatic steatosis and fibrosis biomarkers in nonalcoholic steatohepatitis (NASH) patients, but long‐term administration may elevate the risk of cancer [9]. Besides, FGFR inhibitors yield clear clinical benefits for tumors harboring FGFR aberrations. However, erdafitinib, a pan‐FGFR inhibitor, frequently induces dose‐limiting hyperphosphatemia in the treatment of urothelial carcinoma due to concurrent inhibition of the renal FGF23–FGFR1 axis, representing a major safety bottleneck for its clinical application [10]. Additionally, the common FGFR2 kinase domain “gatekeeper” residue V565F mutation in patients with cholangiocarcinoma renders FGFR2‐specific inhibitors ineffective [10]. Collectively, these clinical observations underscore the functional pleiotropy and regulatory complexity inherent in the FGF/FGFR signaling network. Therefore, a comprehensive understanding of the FGF/FGFR signaling is urgently necessary to develop more precise intervention strategies for targeting this signaling.

In this review, we provide an overview of FGF/FGFR signaling, its cellular and physiological functions, and diseases associated with dysregulated FGF/FGFR signaling. Furthermore, we also discuss the strategies targeting this signaling and provide new perspectives for this research field.

## 2 Overview of FGF/FGFR Signaling

**FIGURE 1:**
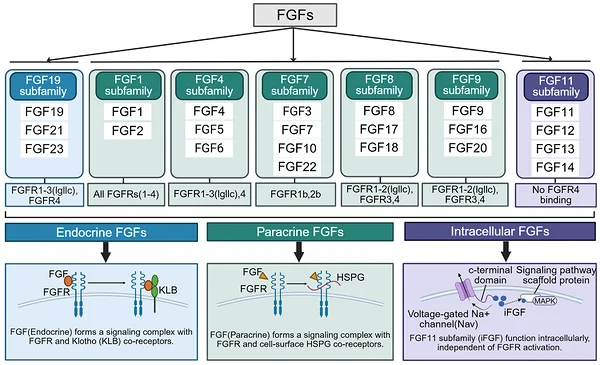
Classification and functional signaling characteristics of the FGF family. Fibroblast growth factors (FGFs) can be divided into three major functional categories according to their structural features, modes of action, and receptor preferences: endocrine FGFs (represented by the FGF19 subfamily), paracrine FGFs (including the FGF1, FGF4, FGF7, FGF8, and FGF9 subfamilies), and intracellular FGFs (iFGFs, viz., the FGF11 subfamily). Endocrine FGFs assemble with FGFR and the specific Klotho (KLB) co‐receptor to form a functional signaling complex. Paracrine FGFs exert their biological functions by forming a signaling complex with FGFR, utilizing cell surface heparan sulfate proteoglycans (HSPGs) as co‐receptors. In contrast, intracellular FGFs (FGF11 subfamily) function independently of FGFR activation and directly act intracellularly to regulate neuronal excitability, MAPK, and other downstream signaling pathways, thereby mediating unique biological effects. Different FGF subfamilies also exhibit differential FGFR binding preferences. Created with Biorender.com.

The FGF/FGFR signaling system comprises FGFs (ligands) and FGFRs (receptors), along with essential regulatory components, including the co‐receptors Klotho (for endocrine FGFs), extracellular FGF binding proteins (FGFBPs), and heparan sulfate proteoglycans (HSPGs), which collectively govern FGF presentation, FGFR activation, signal transduction, and specificity (Figure 1).

### 2.1 The Components of FGF/FGFR Signaling

#### 2.1.1 FGF Family

The human FGF family comprises 22 members, which are classified into five paracrine subfamilies (FGF1, FGF4, FGF7, FGF8, and FGF9), one endocrine subfamily (FGF19), and one intracellular subfamily (FGF11) based on sequence homology, phylogenetic relationships, and secretion modes [11]. This functional classification closely correlates with their distinct mechanisms of receptor engagement and signaling. Paracrine and endocrine FGFs activate FGFRs on the cell membrane to execute their functions, whereas intracellular subfamily FGF11 (also known as intracellular homologous factor) does not bind to FGFRs. Instead, it functions autonomously within the cytoplasm and nucleus, where it modulates ion channel activity. Specifically, FGF11 acts directly within the cell, mainly regulating intracellular voltage‐gated sodium channels and other proteins, thereby participating in regulation of neuronal excitation and cardiac electrical activity [12, 13].

All FGF members are composed of 150–300 amino acids (AAs) and share a conserved core domain of approximately 125 AAs. This conserved core domain adopts a canonical β‐trefoil fold formed by 12 antiparallel β‐strands, which constitutes the key region responsible for receptor binding and exerts its functions [14, 15]. Moreover, this domain contains typical heparan sulfate (HS) binding sites that enable paracrine FGFs to anchor to HSPGs on the cell surface, thereby facilitating the formation of a stable FGF–FGFR–HS ternary complex and the subsequent initiation of intracellular signal transduction [16]. Nevertheless, marked structural and functional divergence exists across FGF subfamilies. Endocrine FGFs lack the β11 strand, leading to an atypical β‐trefoil fold that impairs high‐affinity heparin binding. Consequently, they require the co‐receptor Klotho to bind with FGFR and trigger downstream signal transduction [12]. For instance, endocrine FGFs, including FGF19, FGF21, and FGF23, are secreted into the circulation and act on distal target organs co‐expressing both FGFRs and Klotho co‐receptors [17]. Moreover, the N‐terminal and C‐terminal regions of FGF family members exhibit pronounced sequence divergence, which confer a fundamental molecular determinant of FGF functional diversity [18].

#### 2.1.2 Fibroblast Growth Factor Receptors

As the specific receptor of FGF ligands, FGFRs are a typical class of receptor tyrosine kinases (RTKs) that consist of four highly homologous receptor subtypes (FGFR1–FGFR4) [19]. As single‐pass transmembrane proteins, each FGFR subtype is composed of approximately 800 AA residues [11] and exhibits a conserved domain architecture that includes three immunoglobulin‐like extracellular domains (D1–D3), a hydrophobic transmembrane domain (TMD), and a cytoplasmic tyrosine kinase domain with catalytic activity [20, 21]. Among these domains, the D1 domain, in collaboration with the adjacent acidic box, mediates conformational autoinhibition, thereby effectively preventing receptor dimerization and spontaneous phosphorylation in the absence of ligands. Meanwhile, the D2 and D3 domains collectively form the core ligand‐binding region, in which sequence variations and spatial arrangement directly determine the recognition specificity and binding affinity for distinct FGF ligands [22, 23].

Alternative splicing of FGFR transcripts can precisely regulate the specificity of FGFR signaling. Notably, alternative splicing of the D3 domain in FGFR1–FGFR3 generates two distinct isoforms, designated as IIIb and IIIc [24], and this splicing event exhibits pronounced tissue specificity. Generally, epithelial cells predominantly express the FGFR‐IIIb subtype, which displays high affinity for mesenchymal cell‐derived FGF2 and FGF7. Conversely, mesenchymal cells primarily express the FGFR‐IIIc subtype, which shows high affinity for epithelial cell‐derived FGF8 [25].

In contrast, FGFR4 does not undergo alternative splicing to produce distinct isoforms, and no significant differences in FGF binding affinity have been observed between its long and short variants [26]. Collectively, this finely tuned splicing regulation of FGFRs, together with the tissue‐specific patterns of FGF–FGFR, constitutes a key mechanism for orchestrating the activation of the FGF/FGFR signaling and maintaining normal tissue homeostasis.

#### 2.1.3 Klothos and HSPG

The precision of FGF/FGFR signaling depends on the fine‐tuned regulation of co‐receptor and binding proteins [27]. These accessory molecules enhance ligand–receptor affinity through complex structural mechanisms, modulate the transmission of FGF/FGFR signaling, and ensure the activation of downstream signaling pathways with high binding strength. The two most important types of accessory molecules are the Klotho proteins and HSPGs.

Klotho proteins serve as essential co‐receptors mediating the biological effects of endocrine FGFs (FGF19, FGF21, and FGF23) and primarily comprise two subtypes: α‐Klotho and β‐Klotho [28, 29]. Both are single‐pass transmembrane proteins, and their extracellular regions contain two glycosidase homology domains, designated Klotho homology domain 1 (KL1) and Klotho homology domain 2 (KL2). Throughout evolution, although these two domains have lost their original enzymatic catalytic activity, they have acquired new protein interaction interfaces, thereby gaining the function of co‐receptors [30]. In contrast to paracrine FGFs, which rely on electrostatic interactions with HSPGs for stabilization, endocrine FGFs exhibit markedly reduced heparin‐binding affinity due to conformational alterations in the β11 strand of their core domain. Consequently, endocrine FGFs require Klotho proteins as molecular scaffolds to achieve receptor dimerization [29, 31].

Cryo‐electron microscopy structural analysis has revealed the fine architecture of the FGF–FGFR–Klotho ternary complex. The extracellular domain of the Klotho protein, via its KL2 domain, simultaneously engages the C‐terminus of the FGF ligand and the D3 domain of FGFR, thereby forcibly bringing the receptor and ligand under conditions of low heparin affinity and inducing the formation of a stable signaling complex [32, 33]. This binding mode exhibits extremely high selectivity. For instance, α‐Klotho specifically assists FGF23 in activating FGFR1c to regulate phosphorus metabolism, whereas β‐Klotho mainly assists FGF19 and FGF21 in activating FGFR4 and FGFR1c, respectively, participating in the regulation of BA and lipid metabolism [29, 34]. In addition to functioning as a transmembrane co‐receptor, Klotho family proteins also exhibit remarkable molecular versatility. Their extracellular domains can be cleaved and shed by proteases, such as ADAM10/17, and enter the bloodstream as soluble Klotho (sKlotho). sKlotho is a biologically active form that participates in several critical physiological functions, such as anti‐oxidative stress, maintaining ion channel homeostasis, and delaying cellular senescence [28].

HSPGs are a class of large composite molecules composed of a core protein and covalently linked to HS‐type glycosaminoglycan (GAG) chains [35]. HSPGs are widely distributed on the cell membrane surface and within the extracellular matrix (ECM) of all animal cells. On the basis of their localization and structure characteristics, they can be categorized into three types: membrane HSPGs, the secreted ECM HSPGs, and the secretory vesicle proteoglycan [36, 37]. The molecular weight of HSPG core proteins varies considerably, ranging from approximately 37 to 400 kDa. The binding of HSPGs to their ligands mainly depends on the sulfated HS chains they carry [38]. The sulfation modification of HS chains plays a key role in stabilizing the FGF–FGFR–HS ternary complex and in regulating FGF signal transduction [39, 40]. X‐ray crystallography analysis has revealed the structural characteristics of the ternary complex, confirming that HS acts as a molecular bridge between FGF and the D2 domain of FGFR by simultaneously binding to both. This interaction effectively induces the formation of a stable 2:2:2 dimeric complex and enhances the binding affinity between FGF and FGFR [41].

#### 2.1.4 FGF Binding Proteins

FGFBPs constitute a class of secreted carrier proteins that can bind to specific FGFs, such as FGF1, FGF2, FGF7, and FGF10, with high specificity in a non‐covalent manner, thereby mobilizing locally acting paracrine FGFs from their extracellular storage [42, 43]. The FGFBP family comprises FGFBP1 (also known as heparin‐binding protein, HBP17), FGFBP2, and FGFBP3 (also known as killer‐specific secretory protein, Ksp37). These proteins typically consist of approximately 250 AAs and are secreted carrier proteins with a molecular weight of approximately 25 kDa [42]. They possess two characteristic functional domains: The N‐terminal domain interacts with heparin chains in the ECM, whereas the C‐terminal domain specifically recognizes the β‐tricyclic domain of FGFs [43, 44]. Under physiological homeostasis, the expression levels of FGFBP remain extremely low, and most FGFs are located in the extracelluar matrix. Upon exposure to tissue injury, inflammation, or malignant transformation, FGFBP expression is markedly upregulated. By taking advantage of their higher affinity than the ECM, FGFBPs competitively bind to FGF, forming FGF‐FGFBP complexes that enable FGFs to precisely engage FGFR binding pockets on adjacent or distant cell membranes [44, 45]. For instance, FGFBP1 binds to FGF5 through liquid–liquid phase separation (LLPS), thereby promoting liver repair [46]. Besides, the FGFBP3 protein interacts with endocrine FGFs through its C‐terminus, and thus potentiates the endocrine FGF/FGFR signaling [47].

### 2.2 The Downstream Signaling Cascades of FGF/FGFR Signaling

**FIGURE 2:**
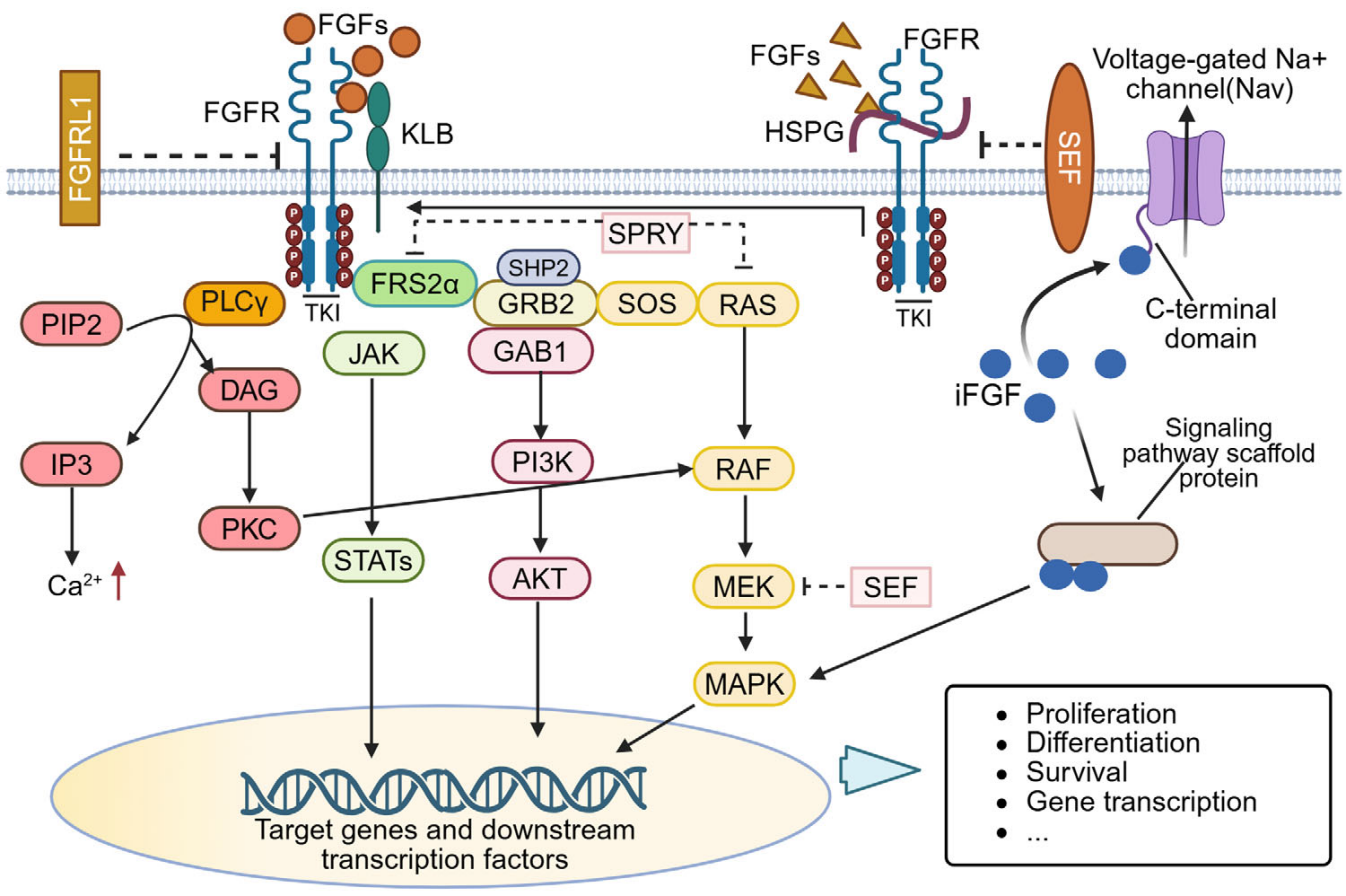
The FGF/FGFR signaling pathway. Following ligand binding and co‐receptor complex assembly, the tyrosine kinase domain of fibroblast growth factor receptor (FGFR) is activated, initiating a series of intracellular signaling cascades. Major downstream signaling axes include the RAS–MAPK, PI3K–AKT, JAK–STAT, and PLCγ‐Ca^2+^ pathways. These cascades ultimately regulate the transcription of target genes and thereby modulate fundamental cellular biological processes such as cell proliferation, differentiation, and survival. Paracrine FGF signaling depends on HSPG co‐receptors, whereas endocrine FGF signaling requires Klotho (KLB) for functional signaling complex formation. The entire signaling pathway is tightly and finely regulated via negative feedback by SPRY, SEF, and FGFRL1. Distinctively, intracellular FGFs (iFGFs) function in an FGFR‐independent manner: They regulate neuronal excitability through voltage‐gated sodium channels and bind to intracellular scaffold proteins to mediate unique biological functions. Mechanistically, SPRY proteins inhibit FRS2–GRB2/SOS‐mediated RAS–MAPK signaling; SEF attenuates FGFR‐proximal and MAPK pathway activation; and FGFRL1 acts as a kinase‐deficient FGFR‐like modulator that can sequester ligands or receptor complexes to limit pathway output. Created with Biorender.com. FGF, fibroblast growth factor; FRS2, fibroblast growth factor receptor substrate 2; GRB2, growth factor receptor‐bound protein 2; HSPG, heparan sulfate proteoglycan; JAK, Janus kinase; MAPK, mitogen‐activated protein kinase; PI3K, phosphatidylinositol 3‐kinase; PLCγ, phospholipase C gamma; RAS, rat sarcoma; SOS, Son of Sevenless; STAT, signal transducer and activator of transcription.

When FGF ligands form a ternary symmetrical complex with FGFR receptors and co‐factors such as HSPG or Klotho proteins, the conformation of the RTK domain changes and undergoes autophosphorylation, which further activates a series of downstream signaling cascades, such as RAS/MAPK, PI3K/AKT, PLCγ/PKC, and STAT pathways (Figure 2).

Upon activation, FGFRs first phosphorylate the fibroblast growth factor receptor substrate 2 (FRS2). Through its phosphotyrosine‐binding (PTB) domain, FRS2α subsequently recruits the growth factor receptor‐bound protein 2 (GRB2)‐Son of Sevenless (SOS) complex, the phosphatase Src homology region 2 domain‐containing phosphatase‐2 (SHP2), and the adaptor protein GRB2‐associated binding protein 1 (GAB1), leading to the activation of two major downstream cascades: the RAS–MAPK (ERK1/2) pathway and the PI3K–AKT–mammalian target of rapamycin (mTOR) pathway [48, 49, 50]. The MAPK pathway primarily regulates gene expression and the cell cycle, whereas the PI3K pathway maintains cell survival and metabolic homeostasis [51]. FGFRs can directly activate PLCγ, which catalyzes the generation of second messengers, induces calcium ion release from the endoplasmic reticulum (ER) and activates the PKC pathway. This cascade modulates key cellular processes, including cell movement, axon guidance, and cytoskeletal remodeling [52]. Additionally, FGFR‐mediated signaling enhances cell adhesion and participates in tissue morphogenesis through interaction with Src family kinases and the Crk/p130Cas pathway. Under stress conditions, FGFR also regulates the balance between cell survival and death by activating the JNK and p38 MAPK pathways [53].

The cascade of signaling events initiated by FGF ligands ultimately converges upon the cell nucleus, where it precisely modulates the expression of downstream target genes through the action of multiple transcription factors, including STATs, activator protein‐1 (AP‐1), and E26 transformation‐specific (ETS). For instance, FGF/FGFR signaling effectively activates the STAT transcriptional pathway (mainly STAT1, STAT3, and STAT5). This activation can be achieved either by direct phosphorylation of STAT tyrosine residues by the kinase domain of FGFR [54, 55] or indirectly amplifying the STAT activation signals via Src family kinases [54]. Once activated, STAT molecules form homodimers or heterodimers and rapidly translocate to the nucleus, where they function as a transcription factor by binding to the promoters of specific target genes, such as *BCL2* and *Cyclin D1* [27]. In the context of metabolic regulation, FGF21 exerts atypical effects by activating the AMPKα pathway, thereby regulating energy balance and lipid metabolism [56, 57, 58].

Additionally, FGF19, secreted by the small intestine, reaches the liver via the portal circulation and binds to the FGFR4/β‐Klotho receptor complex on the surface of hepatocytes. This interaction leads to suppression of cholesterol 7α‐hydroxylase (*CYP7A1*) expression through the activation of the MAPK/ERK/c‐JNK signaling cascade, ultimately reducing BA synthesis [59]. Additionally, FGF23 binds to the FGFR–Klotho complex in the kidney, initiating the MAPK signaling pathway and promoting the internalization of the sodium‐phosphate cotransporters NPT2a and NPT2c on the apical membrane of the proximal epithelial tubules, thereby increasing urinary phosphate excretion [60].

The downstream signaling cascade of FGF/FGFR signaling is governed by stringent, multi‐layered regulatory mechanisms. At the extracellular level, signal amplitude is modulated by the local concentration of FGF ligands, the expression level and spatial distribution of FGFRs, and the extracellular availability of HSPGs [27, 61]. At the intracellular level, negative feedback regulators, including the protein tyrosine phosphatase SHP2 and the lipid phosphatase PTEN, attenuate pathway activity by dephosphorylating key signaling intermediates, thereby preserving signaling homeostasis and preventing hyperactivation [5, 52, 62]. Dysregulation of these control mechanisms, arising from oncogenic *FGF* or *FGFR* gene fusions, amplifications, or epigenetic silencing of feedback inhibitors, can result in constitutive pathway activation. Such aberrant signaling contributes to tumorigenesis or monogenic developmental disorders [63, 64]. In addition, Sprouty (SPRY) proteins restrain RAS–MAPK activation by interfering with FRS2‐GRB2/SOS complex formation [65], and similar expression to fgf genes (SEF) serves as a feedback‐induced antagonist of Ras/MAPK pathway‐mediated FGF/FGFR signaling [66]. Moreover, FGFRL1, a kinase‐deficient FGFR‐like receptor, functions as a decoy or modulatory receptor to dampen excessive FGF/FGFR signaling [67].

In summary, the FGF/FGFR signaling is an evolutionarily highly conserved and functionally complex signaling network. Simply put, it acts like a sophisticated “communication system,” transmitting extracellular signals into the cell through the coordinated action of ligands, receptors, co‐receptors, and accessory factors, thereby precisely regulating cellular functions and physiological activities.

## 3 Functions of FGF/FGFR Signaling

### 3.1 The Role of FGF/FGFR Signaling in Regulating Cellular Functions

**FIGURE 3:**
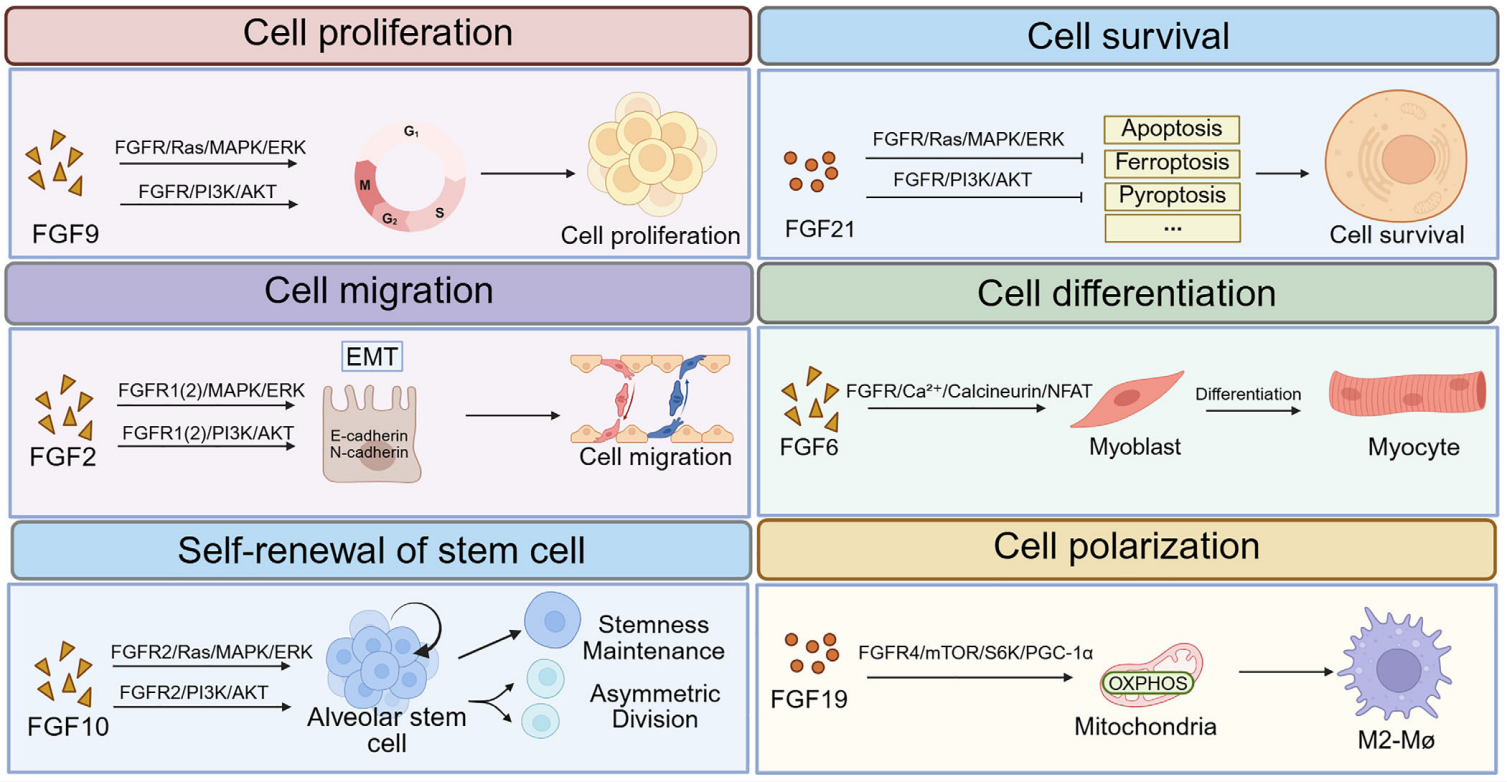
FGF/FGFR signaling regulates multiple cellular biological processes. Different FGF ligands exert extensive regulation on cellular activities by activating specific FGFR downstream signaling cascades. FGF9 activates the MAPK/ERK and PI3K/AKT cascades to accelerate cell cycle progression and promote cell proliferation. FGF21 transmits FGFR‐mediated signaling to suppress various cell death programs, including apoptosis, ferroptosis, and pyroptosis, so as to support cell survival. FGF2 induces EMT through the MAPK/ERK and PI3K/AKT pathways, thereby facilitating cell migration. FGF6 modulates the Ca^2+^/calcineurin/NFAT signaling axis to mediate the differentiation of myoblasts into mature myocytes. FGF10 maintains the stemness and self‐renewal of alveolar stem cells via FGFR2‐dependent signaling. FGF19 regulates mitochondrial oxidative phosphorylation (OXPHOS) through the FGFR4/mTOR/S6K/PGC‐1α pathway, thereby driving M2 macrophage polarization. Created with Biorender.com. EMT, epithelial–mesenchymal transition; M*Φ*, macrophage; MAPK, mitogen‐activated protein kinase; PI3K, phosphatidylinositol 3‐kinase; RAS, rat sarcoma.

By transducing extracellular cues into precisely coordinated intracellular responses via sequential phosphorylation cascades and context‐dependent transcriptional regulation, the FGF/FGFR signaling plays a pivotal role in the regulation of fundamental cellular processes, including cell proliferation, survival, migration, differentiation, polarization, and self‐renewal of stem cells (Figure 3).

#### 3.1.1 Cell Proliferation

Cell proliferation, a fundamental biological process that increases the number of cells in an organism and is achieved through the orderly progression of the cell cycle, is crucial for the growth, development, tissue repair, and regeneration of the organism [68]. FGF/FGFR signaling serves as a pivotal regulator of cell cycle processes and cell proliferation. Central to this effect is the classical RAS–MAPK pathway, wherein activated ERK1/2 phosphorylates diverse transcription factors and cell cycle regulators to facilitate the G1‐to‐S phase transition [50, 69, 70]. However, the impact of FGF/FGFR signaling on cell proliferation is bidirectional, as it can either promote cell cycle progression or induce cell cycle arrest, depending on the specific cell type and physiological context. Multiple FGF/FGFR signalings drive cell cycle progression by activating distinct downstream pathways. In various cancer cells, activating mutations or fusions of FGFR lead to sustained activation of MAPK and PI3K–AKT pathways, thereby endowing tumor cells with unlimited proliferative capacity [71, 72, 73]. During tissue repair, FGF2 binds to FGFR1/2 receptors to activate the Ras–MAPK/ERK signaling pathway, which drives the proliferation and directed migration of fibroblasts, endothelial cells, and keratinocytes. This pleiotropic mitogenic effect significantly promotes wound healing and re‐epithelialization [74, 75, 76]. Similarly, in osteoblasts, FGF2 and FGF8 upregulate the transcription factor runt‐related transcription factor 2 (Runx2) through FGFR1 signaling. Then, Runx2 activates downstream targets such as Osterix, collectively promoting osteoblast proliferation and differentiation and contributing to osteogenesis [77, 78].

Conversely, FGFR3 plays a negative regulatory role in bone development. It exerts an inhibitory effect on bone development by initiating the STAT1 signaling cascade. Activation of the FGFR3‐STAT1 signaling pathway leads to upregulated *p21* expression, which arrests chondrocytes at the G1 phase of the cell cycle, ultimately suppressing the process of endochondral ossification [79, 80, 81].

#### 3.1.2 Cell Survival

Cell survival is a fundamental cellular process essential for tissue homeostasis, development, and stress adaptation. It is governed by tightly regulated molecular mechanisms, including anti‐apoptotic signaling, regulated necrosis, and cytoprotective autophagy [82].

FGF2 possesses cardioprotective functions. Studies have shown that FGF2 exerts cardioprotective effects by activating the MAPK and PI3K–AKT signaling pathways, inhibiting mitochondria‐ and death receptor‐mediated apoptosis, improving cardiac functional outcomes after ischemia‐reperfusion injury, and maintaining myocardial contractile function [83, 84]. FGF21 enhances cardiac tolerance by binding to the FGFR1/β‐Klotho receptor complex on cardiomyocytes, thereby activating downstream AMPK and PI3K–AKT signaling pathways. This activation subsequently upregulates mitochondrial uncoupling protein 3 (UCP3) and superoxide dismutase 2 (SOD2) expression, curtails reactive oxygen species (ROS) accumulation to attenuate oxidative stress, and suppresses caspase‐3 cleavage, collectively improving cardiomyocyte survival and metabolic adaptation under stress conditions [85]. In the nervous system, FGF2 promotes the development of midbrain dopaminergic neurons via FGFR3‐dependent activation of the MAPK/ERK signaling cascade. In contrast, FGF20 functions as a paracrine survival factor within the adult brain, engaging FGFR1c and activating the PI3K–AKT axis to mediate its effects [86, 87]. Thus, both FGF ligands promote dopaminergic neuron survival via spatially and temporally distinct signaling mechanisms. Consistent with these findings, in vitro studies using primary embryonic midbrain dopaminergic neuron cultures have demonstrated that FGF2 induces robust upregulation of transforming growth factor‐beta (TGF‐β), which, in turn, mediates potent neurotrophic and anti‐apoptotic effects [88]. In renal epithelial cells, FGF1 and its engineered heparin‐binding–deficient variant FGF1^ΔHBS^ exert renoprotection through PI3K–AKT activation, with consequent suppression of oxidative injury and production of pro‐inflammatory cytokines [89].

Additionally, ferroptosis is a type of programmed cell death that is iron‐dependent and characterized by the accumulation of lipid peroxides [90]. The FGF/FGFR signaling plays a significant role in the regulation of ferroptosis. For instance, FGF12 protects cardiomyocytes from doxorubicin‐induced ferroptosis through activation of the FGFR1/AMPK/nuclear factor erythroid 2‐related factor 2 (Nrf2) signaling pathway, an effect that limits cardiac injury [91]. In addition, FGF2 alleviates ischemia‐reperfusion injury by activating the Krüppel‐like factor 2 (KLF2)‐Nrf2 signaling axis in the microvascular system, thereby inhibiting ferroptosis in microvascular endothelial cells and reducing microvascular dysfunction [92], further illustrating the context‐dependent anti‐ferroptotic role of FGF/FGFR signaling.

Besides, pyroptosis is a lytic and pro‐inflammatory form of programmed cell death mediated by the gasdermin family of proteins, playing a crucial role in infection immunity, sterile inflammation, and organ damage [93]. In acute kidney injury (AKI), FGF23 confers protection through induction of FGFR4‐dependent autophagy, an event that antagonizes gasdermin E (GSDME)‐mediated pyroptosis and preserves renal function [94]. Overall, FGF/FGFR signaling enhances cellular viability by suppressing multiple cell death pathways, such as apoptosis, ferroptosis, and pyroptosis.

#### 3.1.3 Cell Migration

Cell migration refers to the process by which cells move directionally from their original location to a target location. This dynamic process plays a crucial role in diverse biological processes, including embryonic development, tissue repair, inflammatory responses, and tumor cell dissemination [95]. Accumulating evidence has demonstrated that the FGF/FGFR signaling is involved in regulating cell migration across multiple cell types. For instance, in keratinocytes, FGF2 drives wound‐edge cell migration and re‐epithelialization by upregulating epithelial–mesenchymal transition (EMT)‐related transcription factors, including Snai2, alongside TGF‐β pathway‐associated genes. Consequently, epithelial adhesion molecules are downregulated, mesenchymal markers upregulated, and β‐catenin translocates to nucleus, thereby promoting wound healing [76]. Additionally, FGF7 binds to keratinocyte‐specific receptor FGFR2IIIb, activating the MAPK/ERK and PI3K/AKT cascades and concurrently driving the proliferation and migration of keratinocytes and facilitating wound re‐epithelialization [96, 97]. Besides, in myoblasts, FGF2 and FGF6 significantly enhance chemotactic migration by synergistically promoting adhesion to the ECM and facilitating cytoskeletal reorganization [98]. Furthermore, FGF13 stabilizes the microtubule network in neurons through direct binding with microtubules, simultaneously exerting dual regulatory functions that promote polymerization and inhibit depolymerization, thereby driving neuronal polarization and migration [99].

#### 3.1.4 Cell Differentiation

Cell differentiation refers to the process by which unspecialized cells acquire specific forms and functions during development, serving as the foundation of embryonic development, tissue formation, and organ function establishment [100]. This process involves changes in cell morphology, physiological characteristics, and other cellular features, enabling cells to perform distinct biological functions. During bone development, FGF9 binds to the FGFR3c and activates the MAPK/ERK signaling pathway, thereby modulating the differentiation of early hypertrophic chondrocytes [101]. Moreover, FGF6 triggers the Ca^2+^/calcineurin/nuclear factor of activated T cells (NFAT) cascade in myoblasts, a signaling axis that upregulates myogenic determinants, including MYOG, to commit cells to differentiation [102]. Meanwhile, FGF20 drives the differentiation of inner ear progenitors through FGFR1–MEKK4‐mediated upregulation of Sox2 and Atoh1, two transcription factors essential for sensory cell specification [103].

#### 3.1.5 Self‐Renewal of Stem Cells

Self‐renewal is the ability of stem cells to maintain the characteristics of stem cells in at least one of their daughter cells during cell division, representing a key attribute that sustains the proliferative capacity of stem cell populations [104]. FGF/FGFR signaling promotes the self‐renewal potential of various adult stem cells by regulating multiple signaling pathways [105]. In nephron progenitor cells, FGF9 and FGF20 bind to FGFR1c and transduce signals through the Ras–MAPK/ERK cascade to elevate the expression of Six2, a transcription factor that maintains stemness. This regulation maintains the stemness characteristics of metanephric mesenchymal stem cells while promoting their differentiation into nephron lineages [106]. Additionally, FGF10 binds to the FGFR2b receptor to activate the PI3K/AKT and Ras–MAPK/ERK pathways, thus modulating the expression balance of Sox9 and Sox2 in alveolar epithelial progenitor cells [106]. This mechanism maintains the pool of distal progenitor cells and drives both the homeostasis and regeneration of alveolar epithelial cells after injury.

With age, aging is accompanied by a marked decline in FGF2 expression within hippocampal astrocytes, thereby leading to attenuation of FGFR1/MAPK/ERK signaling. Consequent to this signaling attenuation, neural stem cells undergo proliferative arrest and precocious differentiation, compromising their self‐renewal potential [107]. During the regeneration process following muscle injury, 11,12‐epoxyeicosatrienoic acid (11,12‐EET) amplifies FGF/FGFR signaling by enhancing the phase separation of FGF ligands on the membrane surface, a process that promotes the proliferation and differentiation of muscle stem cells (MuSCs) [108].

#### 3.1.6 Cellular Polarization

Cellular polarization refers to the establishment of intracellular and functional asymmetry necessary for fulfilling specialized biological roles, representing a critical process in various physiological and pathological contexts [109]. Increasing evidence highlights that FGF/FGFR signaling orchestrates cell polarization in diverse conditions. Specifically, in the neuroectoderm, FGFR1 directly interacts with the core planar cell polarity (PCP) protein Vangl2 and phosphorylates its N‐terminal tyrosine residues. This mechanism is essential for the establishment of PCP and underpins critical morphogenetic events, including neural tube closure [110]. Recently, our team has found that FGF19/FGFR4 signaling drives macrophages toward M2 polarization via activation of the mTOR/ribosomal protein S6 kinase (S6K)/peroxisome proliferator‐activated receptor gamma coactivator 1‐alpha (PGC‐1α) pathway, which enhances the immunosuppressive activity of macrophages [111]. Similarly, FGF21 promotes the conversion of microglia from the pro‐inflammatory M1 phenotype to the anti‐inflammatory M2 phenotype via the SIRT1/NF‐κB pathway, exerting neuroprotective effects [112]. Collectively, these findings underscore the multifaceted roles of FGF/FGFR signaling in orchestrating polarization in development and immune regulation contexts.

### 3.2 The Role of FGF/FGFR Signaling in Regulating Physiological Functions

**FIGURE 4:**
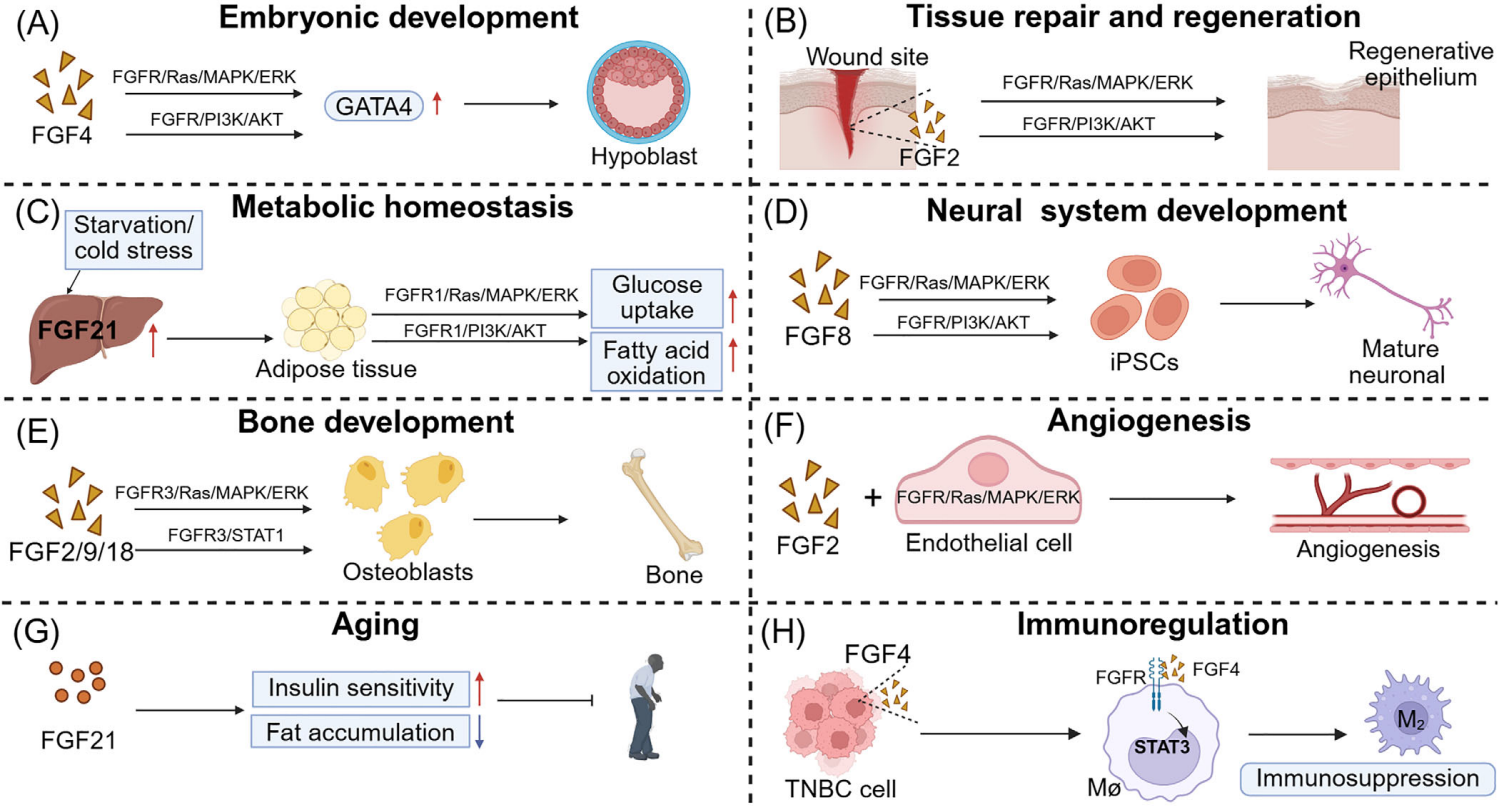
Effects of FGF/FGFR signaling on physiological functions in vivo. FGF/FGFR signaling is widely involved in various biological processes. (A) During embryonic development, FGF4 upregulates GATA4 expression to regulate hypoblast formation. (B) In tissue repair and regeneration, FGF2 at wound sites promotes the remodeling of regenerative epithelium. (C) Under starvation and cold stress, liver‐secreted FGF21 acts on adipose tissue to enhance glucose uptake and fatty acid oxidation, thereby maintaining systemic metabolic homeostasis. (D) During neural system development, FGF8 induces the differentiation of induced pluripotent stem cells into mature neurons. (E) In bone development, FGF2, FGF9, and FGF18 promote osteoblast maturation and bone formation. (F) FGF2 activates endothelial cells to mediate angiogenesis. (G) In the regulation of aging, FGF21 improves insulin sensitivity and reduces fat accumulation to alleviate aging‐related disorders. (H) In the tumor immune microenvironment, FGF4 secreted by TNBC cells induces M2 macrophage polarization by activating STAT3, leading to immunosuppression. Created with Biorender.com. iPSCs, induced pluripotent stem cells; MAPK, mitogen‐activated protein kinase; PI3K, phosphatidylinositol 3‐kinase; RAS, rat sarcoma; TNBC, triple‐negative breast cancer.

By precisely regulating a series of key cellular behaviors, such as cell proliferation, survival, migration, invasion, and differentiation, FGF/FGFR signaling exerts a multidimensional influence over the physiological functions of the organism. It not only orchestrates fundamental life processes, such as embryonic development, tissue repair, immune regulation, and aging, but also plays an integral role in maintaining systemic metabolic homeostasis [5, 113, 114] (Figure 4).

#### 3.2.1 Embryonic Development

Embryonic development is a process by which a single fertilized egg transforms into a complex multicellular organism, relying on a series of precise and complex regulatory mechanisms [115]. During this process, FGF/FGFR signaling guides the morphogenesis and development of various organs through distinct spatiotemporal expression patterns. In limb development, FGF8, secreted by the apical ectodermal ridge (AER), and FGF10, derived from the mesenchyme, form a positive feedback loop through FGFR2b. FGF10 induces FGF8 expression in the AER, whereas FGF8 sustains FGF10 expression in the mesenchyme, thereby driving limb bud initiation, continuous outgrowth, and the formation of the proximodistal pattern [116, 117, 118]. FGFR1 promotes mesenchymal cell survival and proximodistal elongation in the early limb bud, thus driving limb initiation. In contrast, deficiency of FGFR1 leads to mesenchymal cell apoptosis, abnormal AER formation, and downregulation of Sonic hedgehog (Shh) expression, severely disrupting the limb initiation process [119, 120]. In respiratory system development, the FGF10–FGFR2b signal drives the expression of a cohort of development‐related genes through the β‐catenin/EP300 transcriptional complex. This mechanism maintains the stemness of distal epithelial progenitor cells and regulates cell adhesion and rearrangement, thereby driving the repeated branching morphogenesis of the bronchial tree [121, 122, 123].

Additionally, FGF9 and FGF18, derived from mesothelial and epithelial cells, respectively, cooperate to regulate lung parenchymal expansion and alveolarization through actions on distinct mesenchymal subregions. FGF9 participates in WNT/β‐catenin signaling to regulate smooth muscle differentiation, whereas FGF18 upregulates mesenchymal genes such as *SOX9* via FGFR3 and contributes to epithelial branching [124, 125, 126, 127]. In kidney development, FGF10 secreted by the metanephric mesenchyme binds to FGFR2b at the tip of the ureteric bud, a receptor–ligand interaction that stimulates MAPK/ERK signaling to drive ureteric bud branching and elongation. In parallel, in response to signals from the ureteric bud tip, FGF9 and FGF20 secreted by the cap mesenchyme bind to FGFR1/FGFR2 within the mesenchyme, leading to MAPK/ERK‐mediated upregulation of Six2. This mechanism maintains the stemness of nephron progenitor cells and drives their differentiation toward the nephron lineage [128, 129, 130]. Concurrently, FGF8, originating from the mesoderm, inhibits BAX/BAK‐mediated mitochondrial apoptosis to sustain the survival of nephron progenitor cells. Working together with WNT4, FGF8 upregulates Lhx1 to initiate renal tubule differentiation, a step that propels the formation of nascent nephrons [130].

In addition, multiple FGF ligands, such as FGF3, FGF8, FGF10, and FGF20, are involved in distinct stages of inner ear development. At the onset of otic placode induction, endodermal FGF8 triggers FGF19 expression in the adjacent mesoderm. FGF19 then signals the neuroectoderm, where it upregulates WNT8c and FGF3, which together initiate otic placode formation [131]. At the otic vesicle stage, FGF3 and FGF10 bind to FGFR2b to maintain the survival of neural progenitor cells within the otic vesicle and regulate the formation and proliferation of epithelial patterns; these processes coordinate the morphogenesis of the cochlea and vestibule [132]. FGF20, under the regulation of notch signaling, promotes the differentiation of the prospective sensory epithelium into hair cells during sensory hair cell differentiation [103]. Meanwhile, FGF8 secreted by inner hair cells regulates the differentiation of local supporting cells into pillar cells [133]. In the developing nervous system, gradient signals composed of FGF8 and FGF17 regulate the proliferation and differentiation of the midline structures in the cerebellum, ultimately impact cerebellar architecture and function [134].

#### 3.2.2 Tissue Repair and Regeneration

Tissue repair refers to the biological process by which the body restores the structural integrity and function of injured tissues through multiple ordered stages, such as blood coagulation, inflammation, and cell proliferation [135]. Unlike embryonic development, tissue repair requires the rapid mobilization of diverse cellular responses triggered by injury signals, and FGF/FGFR signaling is extensively involved in this injury response and repair process [136]. Following skin injury, FGF2 expression increases rapidly, leading to the activation of FGFR–MAPK signaling in endothelial cells and promoting angiogenesis. Simultaneously, FGF2 drives fibroblast proliferation and collagen deposition for granulation tissue formation, while also promoting keratinocyte migration and proliferation to expedite epithelial regeneration [74, 137]. Collectively, these actions accelerate wound healing. In contrast, although FGF7 regulates hair follicle development by binding to the FGFR2b receptor on keratinocytes, its function in skin wound repair can be partially compensated by FGF10, demonstrating a certain degree of functional substitutability [138].

During skeletal muscle regeneration, FGF6 activates the ERK1/2 signaling pathway via FGFR1 and FGFR4 to upregulate myogenin expression, which subsequently drives myoblast differentiation and promotes muscle repair [102]. Besides, FGF2 and FGF6 jointly activate the MAPK/ERK and PI3K/Akt pathways in muscle stem cells, synergistically promoting myoblast migration. The concurrent absence of both ligands leads to migration defects and hinders the muscle regeneration process [98]. In response to pressure overload, FGF2 secreted by cardiomyocytes activates downstream pathways, such as MAPK, to induce adaptive cardiac hypertrophy [139]. Furthermore, cardiac‐specific overexpression of FGF2 enhances the MAPK signaling cascade, thus alleviating myocardial damage evoked by ischemia‐reperfusion injury [83, 84].

In addition, FGF/FGFR signaling also plays a crucial role in liver regeneration, which refers to the capacity of the remaining liver tissue to restore its original structure and function after partial resection or injury [140]. During this process, FGF15/19 (FGF15 in mice, FGF19 in humans) secreted by the intestine acts on the liver via the portal circulation. Through binding with FGFR4/KLB complex, FGF15/19 signaling activates the MAPK/ERK and PI3K/AKT pathways, driving hepatocyte proliferation and tissue repair [141, 142]. In lung injury models, FGF2 mitigates injury by preserving the integrity of the alveolar epithelial barrier. Meanwhile, FGF10 exerts dual protective effects: It suppresses the NF‐κB pathway to produce anti‐inflammatory actions in the early stage and inhibits the TGF‐β/Smad pathway to mediate anti‐fibrotic effects in the late stage. Collectively, these coordinated actions alleviate bleomycin‐induced pulmonary fibrosis [143, 144].

#### 3.2.3 Metabolic Homeostasis

Metabolic homeostasis refers to the body's ability to maintain relatively constant metabolic parameters through fine‐tuned regulation in response to changes in internal and external environments [145]. The maintenance of this balance requires the coordinated cooperation among different tissues and organs, in which endocrine FGFs serve as important signaling molecules that connect these organs. Especially, endocrine FGFs (FGF15/19, FGF21, and FGF23) form a unique systemic metabolic regulatory network. Acting as hormones, they travel through the bloodstream to distant target organs, where they maintain energy and material metabolic balance [146].

FGF21 is mainly synthesized in the liver. Under conditions of hunger or cold stress, the liver upregulates FGF21 expression via peroxisome proliferator activated receptor (PPAR)‐α. Upon reaching adipose tissue, FGF21 binds to the β‐Klotho/FGFR1c receptor complex to promote glucose uptake and lipolysis. At the same time, it enhances fatty acid oxidation (FAO) and ketogenesis in the liver, jointly maintaining the energy homeostasis [147, 148, 149, 150]. FGF19 is produced by ileal cells upon stimulation of BAs and enters the liver through the portal circulation. It then binds to the FGFR4/β‐Klotho complex on the hepatocytes, inhibiting the expression of CYP7A1, thereby negatively feedback inhibiting the de novo synthesis of BAs. These actions form together a negative feedback loop of BA homeostasis governed by the gut–liver axis [141].

In terms of mineral metabolism, FGF23 secreted by osteocytes is a key regulatory factor for phosphate and vitamin D homeostasis. Upon binding to the FGFR–Klotho complex at the distal convoluted tubule, FGF23 reduces the expression of the sodium‐phosphate cotransporters NaPi‐IIa and NaPi‐IIc, an effect that promotes phosphate excretion into the urine. Simultaneously, it also inhibits CYP27B1 expression, reducing the production of active vitamin D. Together, these actions jointly maintain the stability of blood phosphate levels [151, 152, 153]. Additionally, paracrine FGF1 is activated in adipose tissue under the transcriptional regulation of PPARγ. It facilitates adaptive remodeling of adipose tissue and augments mitochondrial oxidative phosphorylation (OXPHOS) activity via the FGFR1‐ERK1/2 signaling pathway, consequently improving the systemic insulin sensitivity. Collectively, these findings provide potential targets for therapeutic intervention in metabolic diseases [154].

#### 3.2.4 Neural System Development

In addition to maintaining the body's metabolic homeostasis, FGF/FGFR signaling also plays a crucial regulatory role in neural system development. During the formation of synapses, FGF22 binds to presynaptic FGFR1b/FGFR2b receptors, which activates PI3K‐dependent signaling cascades that drive excitatory synaptic vesicle aggregation and subsequent assembly of functional excitatory synapses. In contrast, FGF7 functions through FGFR2b receptor engagement, where it diminishes inhibitory synaptic vesicle aggregation while simultaneously inhibiting synapse development [155]. Together, these two FGF ligands establish the balance of excitation and inhibition within local neural circuits.

For the development and maintenance of dopaminergic neurons within the substantia nigra, both FGF2 and FGF10 interact with FGFRs expressed on neuronal membranes, activating the PI3K/AKT and MAPK/ERK signaling pathways while suppressing mitochondrial‐mediated apoptosis to provide trophic support. Notably, FGF2 even reduces neuronal death in a 6‐hydroxydopamine injury model, demonstrating its potential neuroprotective effects [86].

It is worth noting that FGF11–FGF14, as non‐secreted intracellular proteins, modulate voltage‐gated sodium (Nav) channel inactivation kinetics along with axonal initial segment positioning via direct C‐terminal of Nav channel interactions. This interaction reduces overall neuronal excitability and influences action potential initiation [156, 157, 158]. For instance, loss of FGF14 leads to abnormal inactivation kinetics of Nav channels in cerebellar Purkinje cells, characterized by reduced membrane excitability and impaired firing rates; these electrophysiological alterations manifest as motor coordination deficits and ataxia phenotypes. In addition, FGF14 deficiency induces changes in the electrophysiological discharge pattern of hippocampal CA1 pyramidal neurons and compromises long‐term potentiation mechanisms at Schaffer collateral‐CA1 synapses. These disruptions contribute to spatial learning and memory deficits [159, 160]. Meanwhile, FGF13 regulates neuronal polarization and migration through its microtubule‐binding domain, which polymerizes tubulin and stabilizes microtubules [99].

#### 3.2.5 Bone Development

Bone development and homeostasis are crucial for the proper functioning of the organism. As a key regulatory axis, FGF/FGFR signaling plays a multifaceted role in the skeletal system, not only promoting bone growth and development during the embryonic stage but also participating in maintaining bone homeostasis in adulthood.

During bone development, multiple FGF ligands exert distinct yet coordinated effects on chondrocytes. For instance, FGF2 inhibits excessive chondrocyte proliferation through activation of the STAT1 signaling pathway. FGF9 binding to FGFR3 induces MAPK and PI3K/AKT pathway activation, resulting in suppressed osteogenesis and enhanced osteoclast formation. Besides, FGF18, also through FGFR3 binding, upregulates *Sox9* expression via activating the MAPK/ERK and STAT1 pathways. Through the above pathways, FGF18 inhibits excessive chondrocyte proliferation, while promoting their differentiation and matrix synthesis. Collectively, these three factors coordinately modulate chondrocyte proliferation, hypertrophy, and vascular invasion in long bones, thereby sustaining longitudinal bone growth [101, 161, 162]. Additionally, FGF23/FGFR1c signaling facilitates urinary phosphorus excretion and diminishes production of activated vitamin D, contributing to phosphorus homeostasis and indirectly regulating bone mineralization [163, 164].

Among FGFRs, FGFR3 serves as a critical negative regulator of chondrocyte proliferation in the growth plate. Gain‐of‐function mutations (such as G380R and G382D) lead to ligand‐independent, autonomous activation of FGFR3. This persistent activation continuously stimulates ERK signaling, which suppresses autophagy while promoting the HSPB6‐mediated ferroptosis pathway, ultimately eroding the stemness properties of resting zone chondrocytes and resulting in achondroplasia (ACH). Conversely, FGFR3 deficiency relieves this inhibition, leading to excessive bone growth [165, 166, 167]. Moreover, studies have demonstrated that FGFR3 deficiency leads to upregulation of matrix metalloproteinase‐13 (MMP13) and type X collagen expression in articular cartilage. This causes excessive degradation of proteoglycans and Type II collagen, chondrocyte hypertrophy, and diminished mechanical properties of articular cartilage, ultimately resulting in early arthritis‐like lesions and decreased bone density. These findings indicate that FGFR3 plays an important and sustained role in maintaining adult bone and joint health [168, 169].

#### 3.2.6 Angiogenesis

Angiogenesis refers to the process of generating new blood vessels from the existing vascular network through the proliferation, migration, and lumen formation of endothelial cells, serving as an important basis for vascular system remodeling and expansion [170]. Within the regulatory network of angiogenesis, FGF/FGFR signaling is a key factor in both maintaining vascular homeostasis and inducing pathological angiogenesis.

FGF/FGFR signaling contributes to the maintenance of vascular tone and endothelial homeostasis. For example, FGF2 activates the FGFR‐ERK signaling pathway in endothelial cells, which then stimulates endothelial nitric oxide synthase (*eNOS*) expression via the AP‐1 transcription factor, enhancing nitric oxide (NO) biosynthesis and resulting in vasodilation [171]. Furthermore, activation of FGFR1 sustains homeostatic balance in endothelial cells by antagonizing TGF‐β/Smad signaling, which inhibits endothelial–mesenchymal transition (EndMT) and protects vascular function [172]. Following injury stimulation, FGFR1/2 on endothelial cells drives endothelial cell proliferation and migration through MAPK signaling cascade, facilitating neovascularization in response to tissue [173]. During angiogenesis, FGF2 directly activates the FGFR–MAPK pathway in endothelial cells to promote their proliferation and lumen formation, while simultaneously recruiting NG2+ pericytes and F4/80+ macrophages from bone marrow to build a pre‐vascular network. Subsequently, CD31+ endothelial cells infiltrate and cover this network, leading to the formation of mature blood vessels [174].

In addition to its roles under physiological conditions, FGF/FGFR signaling participates in the initiation and progression of various vascular diseases under pathological conditions. Within atherosclerotic plaques, FGF2 induces endothelial cell proliferation and migration by activating the FGFR–MAPK signaling and augments macrophage infiltration into the plaque. These actions drive intra‐plaque neovascularization and increase blood flow supply within the plaque [175, 176]. Additionally, abnormally elevated FGF23 activates the FGFR‐ERK and NF‐κB pathways in vascular cells and upregulates the synthesis of sulfated glycosaminoglycans (sGAG) and hyaluronic acid (HA). Subsequently, increased concentrations of sGAG and HA remodel the ECM and promote vascular calcification, hastening arteriosclerosis progression. Concurrently, FGF23 can directly act on cardiomyocytes to induce pathological myocardial hypertrophy [177, 178].

Within the tumor microenvironment (TME), FGFBP1 enhances the FGF2–FGFR1‐ERK1/2 signaling axis, which activates *FAPα* expression in hepatic stellate cells (HSCs). Then, activated HSCs promote EMT and the recruitment of immunosuppressive cells through the C‐X‐C motif chemokine ligand 5 (CXCL5)/C‐X‐C motif chemokine receptor 2 (CXCR2) signaling pathway. Consequently, these cascades mediate vascular co‐option and resistance to anti‐angiogenic therapy [179]. Furthermore, FGF2 upregulates platelet‐derived growth factor receptor (PDGFR) expression in endothelial cells, whereas platelet‐derived growth factor‐BB (PDGF‐BB) upregulates FGFR1 expression in mural cells, forming a bidirectional positive feedback loop that cooperatively promotes tumor angiogenesis and metastasis [180].

#### 3.2.7 Aging

Aging is a progressive process characterized by the decline of tissue function and regenerative capacity, resulting from the combined effects of intrinsic and extrinsic factors [181, 182]. Emerging evidence indicates that FGF/FGFR signaling plays a pivotal role in regulating stem cell aging, tissue aging, as well as systemic aging.

FGF/FGFR signaling regulates the fate of adult stem cells through multiple mechanisms. For instance, in bone marrow mesenchymal stem cells (BMSCs), the effect of FGF2 on BMSC aging is dose‐dependent. Low levels of FGF2 promote BMSC mitotic activity via activation of the AKT and ERK pathways. However, chronic high‐dose FGF2 treatment results in ERK1/2 dephosphorylation, elevated LC3‐II‐mediated autophagy, and enhanced senescence‐associated β‐galactosidase (SA‐β‐gal) activity, ultimately causing BMSCs growth inhibition and accelerated senescence [183, 184]. In adipose tissue, excessive mitochondrial ROS downregulates the expression of *CD90* in adipose stem cells (ASCs), leading to diminished glucose uptake and pentose phosphate pathway activity that culminates in ASC cell senescence. Conversely, FGF21 upregulates CD90 glycosylation and AKT‐GLUT4 axis activity, enhancing glucose influx and antioxidant capacity to ameliorate cellular senescence [185].

Moreover, age‐related dysregulation of FGF pathway can damage organ function. For example, FGF2 signaling is diminished in cranial osteoblasts with age, impairing osteogenic differentiation [177, 186]. Studies have shown that when leptin receptor‐positive bone stem cells undergo aging, leptin activates the STAT3 signaling pathway to upregulate FGF7 transcription. Subsequently, the elevated *FGF7* promotes osteogenic differentiation of bone stem cells and inhibits osteoclastogenesis, leading to abnormal subchondral bone remodeling and accelerating the development of osteoarthritis (OA) [187]. In addition, the expression patterns of FGF2 and FGF7, as well as their receptors, change significantly during wound healing in aged mice, resulting in decreased fibroblast migration ability and delayed wound healing [96]. Additionally, aging reduces the proportion of FGF2‐expressing astrocytes in the hippocampus, diminishing FGFR1–MAPK/ERK signal and ultimately suppressing neural stem cell proliferation and cognitive decline [107].

Within the context of organismal aging, endocrine FGFs participate in systemic metabolic homeostasis and the aging process through their hormone‐like actions. For instance, FGF23 knockout mice exhibit hyperphosphatemia and excessive vitamin D due to upregulated expression of *NaPi2a* and 1α‐hydroxylase in the kidneys, presenting premature aging‐like phenotypes such as growth retardation, osteoporosis, vascular calcification, and multi‐organ atrophy. Notably, restoring FGF23 levels through genetic methods alleviates these premature aging‐like features, indicating that FGF23 delays systemic aging by regulating phosphorus and vitamin D metabolism [188]. Additionally, FGF19 also delays aging by regulating multiple metabolic pathways, reducing the risk of diabetes, obesity, and cardiovascular diseases [189]. KLB, which serves as a co‐receptor of FGF19/FGFR4, has been identified as a longevity protein that can extend lifespan, and its deficiency leads to aging‐like symptoms in humans, including shortened lifespan, skin atrophy, and infertility [190].

#### 3.2.8 Immunoregulation

The immune system is a critical apparatus responsible for executing immune responses in the body. Composed of immune organs, immune cells, and immune molecules, it is tasked with identifying and eliminating foreign antigens, senescent cells, and mutant cells, while maintaining self‐tolerance and the stability of the internal environment [191]. The FGF/FGFR signaling family can influence the immune status of the tissue microenvironment by regulating the interaction between immune cells and stromal cells.

For instance, FGF1 activates the NF‐κB and NFAT pathways by binding to FGFR on the surface of T cells and cooperating with TCR signals, thereby promoting *IL‐2* transcription and subsequently facilitating lymphocyte activation and proliferation [69]. In innate immunity, the role of FGF/FGFR signaling is more complex and even context‐dependent. For example, in rheumatoid arthritis (RA) and psoriasis, FGF2 cooperates with IL‐17 to promote inflammation by synergistically upregulating the expression of *FGFR1* in target cells and enhancing the release of pro‐inflammatory factors like IL‐6 and IL‐8, an amplification loop that reinforces the Th17 cell‐mediated inflammatory response [192]. Conversely, in sepsis models, FGF21 exerts anti‐inflammatory effects by inhibiting the activation of inflammatory pathways in macrophages and promoting their transformation into anti‐inflammatory M2 phenotypes [193].

Furthermore, FGF/FGFR signaling also serves as a key bridge connecting cancer cells, stroma, and immune cells in the TME. For example, tumor‐derived FGF2 has been demonstrated to drive the polarization of tumor‐associated macrophages (TAMs) toward an M2‐like phenotype, accompanied by the secretion of immunosuppressive cytokines, including IL‐10 and TGF‐β [194]. Simultaneously, FGF2 has been shown to impair T cells’ adhesion and infiltration, while also restricting the cytotoxic function of CD8+ T cells, thereby collectively fostering an immunosuppressive TME [194]. In addition, elevated levels of FGF4 derived from triple‐negative breast cancer (TNBC) cells have been reported to activate the IL6/STAT3 signaling axis in macrophages, subsequently promoting their polarization into an immunosuppressive M2 phenotype. As a consequence, these polarized macrophages secrete IL‐10 and TGF‐β, which potently suppress the proliferation of CD8+ T cells, thus contributing to tumor immune escape [195].

Under normal circumstances, the FGF/FGFR signaling is strictly regulated and participates in the regulation of various cellular functions and physiological activities of the body through multiple mechanisms.

## 4 Dysregulated FGF/FGFR Signaling‐Related Diseases

**FIGURE 5:**
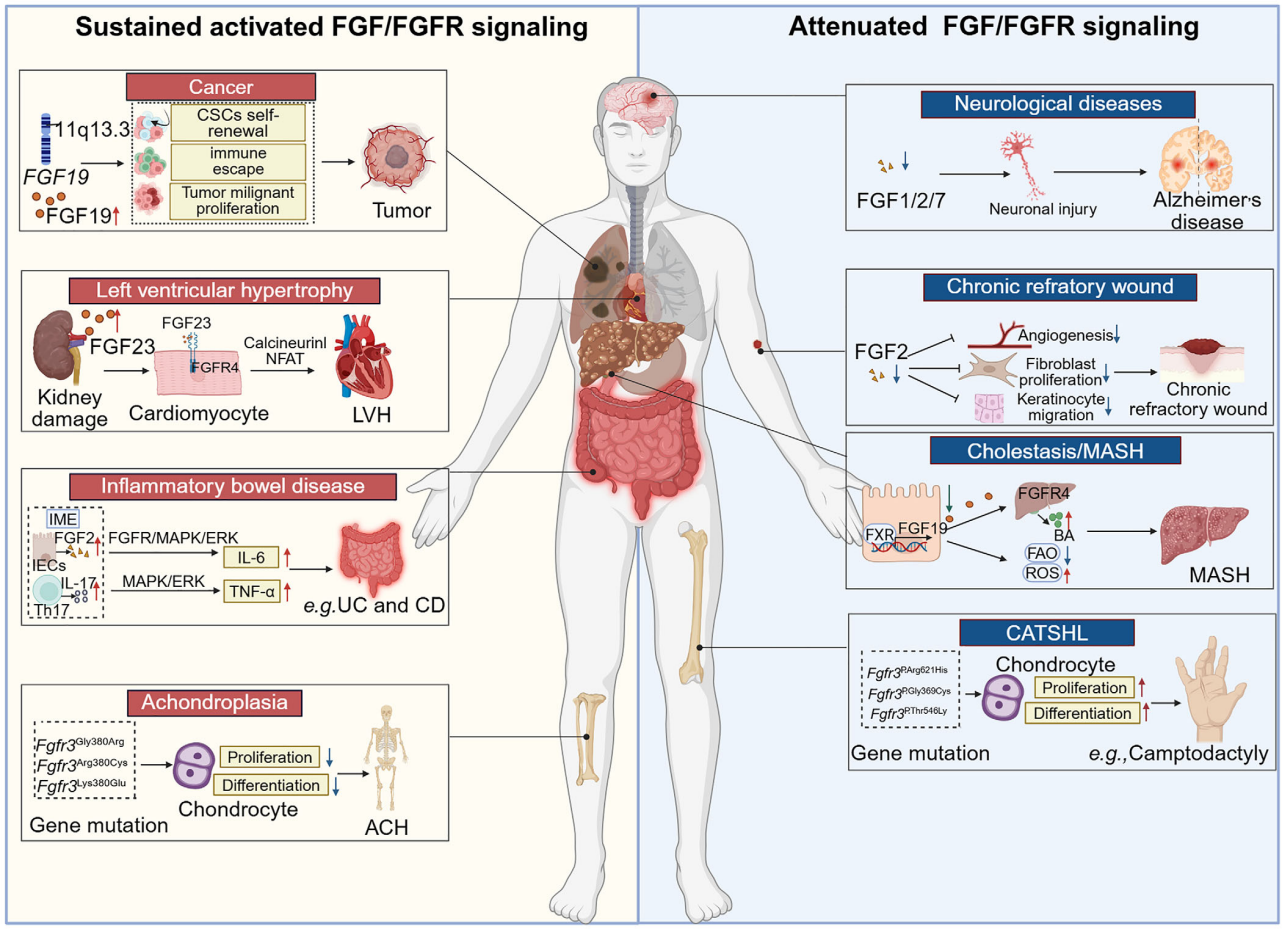
The association between dysregulated FGF/FGFR signaling and multiple human diseases. Aberrant regulation of FGF/FGFR signaling, including sustained overactivation and pathological attenuation, is closely correlated with the occurrence and progression of a wide range of human disorders. Representative diseases corresponding to these two abnormal signaling patterns are illustrated separately as follows. On the left side are disorders associated with sustained excessive activation of FGF/FGFR signaling: Upregulated FGF19 promotes cancer stem cell self‐renewal, immune evasion, and malignant proliferation, thus driving tumor development. Elevated FGF23 after kidney injury acts on cardiomyocyte FGFR4, induces activation of the calcineurin/NFAT pathway, and leads to left ventricular hypertrophy. Increased FGF2 in the intestinal immune microenvironment activates the FGFR/MAPK/ERK and NF‐κB cascades, upregulates pro‐inflammatory cytokines, including IL‐6 and TNF‐α, and aggravates inflammatory bowel diseases such as ulcerative colitis and Crohn's disease. Gain‐of‐function mutations in *FGFR3* inhibit chondrocyte proliferation and differentiation, resulting in achondroplasia. On the right side are diseases related to attenuated FGF/FGFR signaling: Downregulated FGF1/2/7 mediates neuronal injury and contributes to the progression of Alzheimer's disease. Insufficient FGF2 secretion inhibits angiogenesis, fibroblast proliferation, and keratinocyte migration, resulting in chronic refractory wounds. Impaired FGF19/FGFR4 signaling disrupts bile acid and fatty acid metabolism, increases reactive oxygen species production, and induces cholestasis and MASH. In addition, *FGFR3* gene mutations cause insufficient signaling, trigger abnormal cell proliferation and differentiation, and ultimately result in CATSHL syndrome. Created with Biorender.com. ACH, achondroplasia; BA, bile acid; CD, Crohn's disease; FAO, fatty acid oxidation; LVH, left ventricular hypertrophy; ROS, reactive oxygen species; UC, ulcerative colitis.

Under physiological conditions, the transmission of FGF/FGFR signal is precisely regulated. However, multiple factors such as gain or loss‐of‐function mutations in receptors or ligands, gene amplification, the formation of autocrine/paracrine feedback loops, and overexpression of ligands can lead to abnormal FGF/FGFR signaling. Such dysregulation, whether manifesting as overactivation or attenuation, may disrupt the systemic homeostasis and contributes to the pathogenesis of multiple diseases (Figure 5). For instance, activating mutations in *FGFR* serve as key drivers of various cancers, including HCC, cholangiocarcinoma, and bladder cancer [8]. Besides, gain‐of‐function mutations in *FGF23* cause autosomal dominant hypo‐phosphatemic rickets [196], whereas inactivating mutations in *FGFR3* result in excessive bone growth [197].

### 4.1 Sustained Hyperactivated FGF/FGFR Signaling

When FGF/FGFR signaling is hyperactivated, the continuous enhancement of the signal can lead to uncontrolled cell proliferation, abnormal differentiation, increased anti‐apoptotic ability, and so forth, which, in turn, triggers a range of pathological processes, such as metabolic disorders and tumorigenesis.

#### 4.1.1 Diseases of the Skeletal System

ACH is the most common form of hereditary dwarfism in humans, characterized by short limbs, macrocephaly with frontal bossing, lumbar lordosis, and other skeletal deformities [198]. The underlying pathogenic mechanism involves a missense mutation at the 1138th nucleotide of the *FGFR3* gene, where guanine (G) is replaced by adenine (A), resulting in the substitution of a highly conserved glycine residue at position 380 in the TMD of the encoded protein with arginine (G380R) [199]. This mutation enables FGFR3 to form stable dimers spontaneously in the absence of ligands, leading to constitutive activation of the kinase domain [200]. Consequently, it hinders longitudinal bone growth through downstream signaling pathways. First, excessive activation of the MAPK/ERK pathway upregulates p21 expression, which, in turn, arrests chondrocytes in the proliferative zone at the G1 phase of the cell cycle, thereby markedly suppressing their proliferation capacity [81, 201]. Secondly, the mutation disrupts the established Indian hedgehog‐parathyroid hormone‐related protein (IHH‐PTHrP) negative feedback loop within the growth plate, leading to premature hypertrophic differentiation of chondrocytes and dysregulation of their differentiation process [202]. Third, the mutant receptor further intensifies cell cycle arrest through the FRS2‐STAT1 signaling axis [203]. Additionally, mutant *FGFR3* also affects the structure and function of primary cilia in chondrocytes, interfering with Hedgehog and other signaling pathways [204, 205]. Collectively, these alterations impede the longitudinal growth of long bones, leading to short‐limbed dwarfism. In contrast, more severe *FGFR3* mutations (e.g., R248C, K650M/E) that cause lethal dysplasia completely disrupt growth plate architecture and lead to fatal thoracic dysplasia [206, 207].

Craniosynostosis syndrome encompasses a group of congenital craniofacial malformations caused by premature closure of cranial sutures. It is characterized by abnormal head shape, elevated intracranial pressure, midface hypoplasia, exophthalmos and may be accompanied by syndactyly, intellectual disability, and other clinical manifestations [208]. This syndrome is primarily caused by gain‐of‐function point mutations in the *FGFR1*, *FGFR2*, or *FGFR3* genes [209]. Within the context of Apert syndrome, the S252W or P253R mutations in the *FGFR2* gene markedly enhance the receptor's affinity for its ligands and enable aberrant binding to non‐classical ligands [77, 210, 211]. This abnormal ligand–receptor interaction leads to continuous and excessive activation of downstream RAS–MAPK/ERK and PI3K–AKT signaling pathways [212]. A salient consequence of such pathway hyperactivity is the transcriptional upregulation of *Runx2* and *Osterix*, which function as master regulators of osteogenic commitment. This, in turn, accelerates the differentiation of osteoprogenitor cells and promotes pathological matrix mineralization, ultimately resulting in premature fusion of cranial sutures [78]. Mutations in different *FGFR* genes and at distinct sites give rise to varying patterns of cranial suture fusion and associated clinical features. For example, the P252R mutation in *FGFR1* causes Pfeiffer syndrome, whereas the P250R and A391E mutations in *FGFR3* are associated with Muenke syndrome and Crouzon syndrome with acanthosis nigricans, respectively. Collectively, these distinct mutations constitute a complex disease spectrum [213, 214].

OA is the most common degenerative joint disease, characterized by progressive destruction of articular cartilage, subchondral bone sclerosis, osteophyte formation, and synovial inflammation [215]. Studies have demonstrated the FGF/FGFR signaling exerts complex bidirectional regulation in OA. In diseased joints, damaged chondrocytes, activated synoviocytes, and inflammatory cells produce substantial amount of FGF2, which acts through FGFR1, highly expressed on chondrocytes, and in concert with pro‐inflammatory factors such as IL‐1β to activate the MAPK/ERK and PKCδ pathways. This concerted activation, in turn, transcriptionally upregulates *MMP‐13* as well as disintegrin and metalloproteinase with thrombospondin motifs *(ADAMTS)‐4/5*, which mediate the degradation of Type II collagen and aggrecan, the primary structural constituents of cartilage ECM [216]. Conversely, endogenous FGF2 functions as a chondroprotective factor in the destabilization of the medial meniscus (DMM) mouse model of OA, inhibiting ADAMTS‐5 expression and mitigating cartilage degeneration [217]. In contrast, FGF18 mainly transmits anabolic signals via binding to FGFR3 on chondrocytes, promoting the synthesis of proteoglycans and Type II collagen while inhibiting chondrocyte apoptosis [218]. On the basis of this mechanism, recombinant human FGF18 (Sprifermin) has been demonstrated in clinical trials to increase the thickness of knee articular cartilage and slow cartilage loss [219]. Collectively, an imbalance between FGF2‐driven catabolism and FGF18‐driven anabolism is the key driver of the progression of OA.

#### 4.1.2 Metabolic and Endocrine Diseases

X‐linked hypophosphatemic rickets (XLH) is the most common hereditary hypophosphatemic rickets, characterized by rickets in childhood and osteomalacia in adulthood, which is accompanied by hypophosphatemia, renal phosphate wasting, and inappropriately normal or low levels of 1,25(OH)_2_D_3_ [220]. In contrast, tumor‐induced osteomalacia (TIO) is an acquired disorder that typically presents in adults with bone pain, muscle weakness, fractures, and hypophosphatemia, and the symptoms are usually reversible after the surgical removal of the FGF23‐secreting tumor [221]. The core pathophysiological feature underlying both diseases is the abnormally elevated level of FGF23. In XLH, inactivating mutations in the phosphate‐regulating endopeptidase homolog X‐linked (*PHEX*) gene result in impaired degradation of FGF23, leading to elevated circulating concentrations of the phosphatonin [222]. However, benign mesenchymal tumors ectopically produce excessive FGF23 in TIO, resulting in elevated FGF23 levels [223]. Excessive FGF23 mainly binds to the FGFR–Klotho receptor complex located on the apical membrane of renal proximal tubule cells, which activates intracellular signaling cascades that result in the downregulation of sodium‐dependent phosphate transporters NaPi‐IIa and NaPi‐IIc, leading to impaired phosphorus reabsorption. This leads to renal phosphate wasting and hypophosphatemia. Concomitantly, excessive FGF23 inhibits CYP27B1 activity, reducing the synthesis of 1,25(OH)_2_D_3_ and further aggravating the disorder of bone mineralization [224]. Given this pathogenic mechanism, monoclonal antibodies (mAbs) targeting FGF23 have been developed for clinical treatment against XLH [225].

#### 4.1.3 Inflammatory and Autoimmune Diseases

Inflammatory bowel disease (IBD) encompasses a group of chronic and recurrent gastrointestinal inflammatory disorders, characterized by aberrant activation of the intestinal mucosal immune system, leading to inflammation, ulceration, and abnormal repair [226]. Of note, FGF2 level is significantly elevated in the inflamed intestinal mucosa and serum of patients with IBD [227]. Studies have shown a synergistic interaction between FGF2 and IL‐17. Specifically, the upregulation of inflammation‐related genes in intestinal epithelial cells and immune cells is driven by IL‐17 through the activation of the NF‐κB and MAPK signaling cascades. Concurrently, FGF2 activates the MAPK signaling cascade upon binding to FGFR receptors. Together, in coordination with IL‐17‐driven signals, this cascade promotes the expression of pro‐inflammatory cytokines, including IL‐6 and TNF‐α, with the net effect of amplifying local intestinal inflammation. Within this pro‐inflammatory milieu, FGF2 also upregulates tight junction proteins and repairs the intestinal epithelial barrier, suggesting a compensatory protective response mounted by the host in the inflammatory microenvironment [192, 228]. Consequently, targeting FGF2 signaling may represent a potential strategy for regulating inflammation related to the Th17 pathway.

RA is a chronic systemic autoimmune disease characterized by symmetrical and erosive polyarthritis, with the hallmark feature being destruction of articular cartilage and bone resulting from proliferative synovial inflammation [229]. In the synovial tissue of RA, FGF2 level is significantly elevated, primarily due to production by activated macrophages, endothelial cells, and synovial fibroblasts [230]. Within the joint microenvironment, FGF2 synergizes with IL‐17 to engage the ERK signaling axis, a collaborative interaction that robustly induces expression of multiple pro‐inflammatory mediators and chemokines in synovial fibroblasts, including IL‐6, keratinocyte chemoattractant (KC), CXCL2, and cyclooxygenase‐2 (COX‐2) in synovial fibroblasts. The consequence is the amplification of synovial inflammation and the acceleration of joint structural damage [192].

Psoriasis is a prevalent, chronic, and relapsing inflammatory dermatosis pathologically featured with hyperproliferation and aberrant differentiation of epidermal keratinocytes, dermal capillary angiogenesis, and dense inflammatory cell infiltration [231]. Elevated expression of FGF2 has been consistently observed in lesional skin of patients with psoriasis [232]. Analogous to its role in RA, FGF2 acts synergistically with IL‐17 to drive keratinocyte dysregulation, manifesting as both uncontrolled proliferation and enhanced production of pro‐inflammatory mediators. Therefore, FGF2 contributes critically to disease amplification and chronicity [228].

Atherosclerosis is a chronic inflammatory vascular disease driven by the subintimal accumulation of lipids and inflammatory cells, a process that gives rise to atherosclerotic plaques, which can subsequently precipitate vascular stenosis, plaque rupture, and thrombosis [233]. Studies have shown that significantly elevated expression of FGF2 within atherosclerotic plaques is mainly produced by macrophages and vascular smooth muscle cells (VSMCs) residing in the lesions [175]. Upon binding to FGFR1 on VSMCs, FGF2 stimulates their proliferation and migratory activity while simultaneously activating the downstream RAS–MAPK/ERK cascade, contributing to intimal hyperplasia and atherosclerotic plaque progression [234, 235]. Additionally, FGF2 can bind to FGFR on endothelial cells to induce intraplaque neovascularization. However, when acting alone, the neo‐vessels induced by FGF2 are structurally fragile and exhibit insufficient pericyte coverage, rendering them prone to rupture and hemorrhage, which, in turn, increases plaque instability, predisposes to plaque rupture, and promotes thrombosis [176, 236]. Beyond its effects on VSMCs and endothelial cells, FGF2 also promotes macrophage chemotaxis and survival, activates NADPH oxidase to elicit oxidative stress, and amplifies local inflammatory responses [237, 238]. Collectively, these actions position FGF2 signaling as a critical driver of both plaque progression and vulnerability.

#### 4.1.4 Cardiovascular Diseases

Hypertension is a clinical syndrome characterized by persistent elevation of systemic arterial pressure, with its pathophysiology primarily involving an imbalance between vasoconstriction and vasodilation, vascular remodeling, and endothelial dysfunction. It constitutes a major risk factor for cardiovascular and cerebrovascular events [239].

Under pathological conditions, the continuous aberrant expression of FGF2 in local blood vessels promotes VSMC proliferation and migration. Specifically, FGF2 binds to FGFR1 on VSMCs and activates downstream signaling cascades, including the RAS–MAPK/ERK pathway, which upregulates expression of proteins implicated in cell migration. This process contributes to vascular wall remodeling and thickening, indirectly affecting blood pressure regulation [180]. Additionally, abnormally elevated FGF23 in patients with chronic kidney disease (CKD) exerts direct cardiovascular toxicity [177]. High concentrations of FGF23 induce pathological cardiomyocyte hypertrophy by directly activating FGFR4 on cardiomyocytes through a Klotho‐independent mechanism. This signaling involves activation of PLCγ and the downstream calcineurin‐nuclear factor of NFAT pathway. Studies show that FGF23‐induced cardiomyocyte hypertrophy occurs even in the absence of Klotho. This mechanism represents a critical pathological basis for heart failure and cardiovascular death in patients with CKD [178, 240].

Cardiomyocyte hypertrophy represents an adaptive response of the heart to excessive pressure or volume load, but long‐term pathological hypertrophy may progress to heart failure, a condition characterized by a decline in cardiac pumping function that fails to meet the body's metabolic demands [241]. In addition to the direct pro‐hypertrophic effect of FGF23 mentioned above, local FGF/FGFR signaling in the heart is also involved. In a pressure overload‐induced model of cardiomyocyte hypertrophy, cardiac FGF2 expression is upregulated [242]. Paracrine FGF2 released by cardiac fibroblasts binds to FGFR1 on cardiomyocytes and activates MAPK signaling pathway, which drives cardiomyocyte hypertrophy. At the same time, FGF2 activates FGFR1 on cardiac fibroblasts. This receptor–ligand interaction does not merely drive fibroblast proliferation but also promotes their transformation into myofibroblasts and potentiates the production of TGF‐β and collagen. These changes collectively set the stage for myocardial interstitial fibrosis [243, 244]. In contrast to FGF2, FGF21 exerts cardioprotective effects. Under conditions of cardiac stress, FGF21 production is increased [85]. By binding to the FGFR1/β‐Klotho complex, FGF21 activates the PI3K–AKT, AMPK‐SIRT1‐PGC‐1α, and Nrf2 signaling pathways, exerting anti‐apoptotic, anti‐hypertrophic, and metabolic‐improvement effects on cardiomyocytes, while also reducing oxidative stress [85].

#### 4.1.5 Tissue Fibrotic Diseases

Idiopathic pulmonary fibrosis (IPF) is a chronic and progressive interstitial lung disease in which aberrant scarring of the lung parenchyma drives a progressive deterioration of pulmonary function, culminating in hypoxemia and respiratory failure [245]. Studies have demonstrated that FGF/FGFR signaling plays a dual role in IPF. For instance, FGF2 is upregulated in the lung tissue of patients with IPF, where it promotes the proliferation and migration of lung fibroblasts and induces their differentiation into myofibroblasts upon binding to FGFR. Additionally, FGF2 signaling enhances the sensitivity of the TGF‐β pathway, synergistically facilitating the formation and expansion of fibrotic lesions [246, 247]. In contrast, FGF1 and FGF10 exhibit anti‐fibrotic potential. FGF1, for example, suppresses TGF‐β/Smad signaling, with a consequent reduction in myofibroblast differentiation and collagen deposition [248]. Besides, FGF10 primarily impedes fibrosis progression by maintaining the homeostasis and regenerative capacity of alveolar Type II epithelial cells [106].

Renal fibrosis represents the outcome of almost all CKDs progressing to end‐stage renal failure and is histopathologically characterized by the accumulation of activated myofibroblasts in the renal interstitium and excessive deposition of ECM collagen [249]. In the context of renal fibrosis caused by conditions such as diabetic nephropathy and chronic glomerulonephritis, injury to renal tubular epithelial cells leads to increased local FGF2 expression. Upon engagement with FGFR1, FGF2 drives the transformation of renal interstitial fibroblasts/pericytes into myofibroblasts. These activated cells then overproduce ECM components, including collagen, with consequent aggravation of interstitial fibrosis and deterioration of renal function [250]. In contrast, FGF21 has shown renoprotective effects in various animal models of CKD. In detail, FGF21 attenuates glomerulosclerosis and tubulointerstitial fibrosis by inhibiting NF‐κB/NLRP3 inflammasome activation, reducing macrophage infiltration, and decreasing the production of pro‐fibrotic factors, including TGF‐β1 and connective tissue growth factor (*CTGF*) [251]. Additionally, FGF1 also alleviates diabetic nephropathy damage through its anti‐inflammatory and antioxidant properties [252]. Building upon these findings, researchers have developed an FGF1 variant (FGF1^ΔHBS^), which upregulates PPARγ expression, enhances the interaction between PPARγ and SMAD3, and inhibits SMAD3 nuclear translocation in CKD mouse models [89]. This FGF1 variant further suppresses TGF‐β1/Smad3‐driven EMT, diminishing podocyte depletion and renal fibrosis [91].

#### 4.1.6 Cancer

Cancer is a type of clonal neoplasm driven by abnormal genetic or epigenetic alterations in cells and represents the second leading cause of death worldwide [253]. Aberrant activation of FGF/FGFR signaling has been observed in a variety of malignant tumors, mainly driven by the dysregulated expression of FGF/FGFR, activating mutations of *FGFR*, and aberrant KLB receptors (Table 1). Aberrant activation of FGF/FGFR signaling constitutes a crucial oncogenic mechanism across multiple types of cancer, influencing tumor initiation, progression, and therapeutic response.

**TABLE 1:** Mechanisms of fibroblast growth factor (FGF)/FGF receptor (FGFR) signaling aberrations in human cancers.

Aberration type	Involved FGF/FGFR members	Effects	Signaling pathway	Tumors	References
Ligand/receptor overexpression	FGF2	Invasion, migration, angiogenesis	MAPK, PI3K/AKT, PLCγ	NSCLC, CRC	[64, 254]
	FGF4	Proliferation, survival, invasion	STAT3, MAPK, PI3K/AKT	TNBC	[195]
	FGF8	Proliferation, invasion, migration, angiogenesis	MAPK, PI3K–AKT, YAP1, PLCγ, JAK–STAT	PCa, BC, CRC, OSCC, HCC, ARMS	[255, 256, 257, 258, 259, 260]
	FGF19	Proliferation, anti‐apoptosis, metabolic reprogramming	RAS–MAPK, PI3K/AKT, GSK3β/Nrf2, SOCE/NFATc2, PLCγ, JAK–STAT	HCC	[72, 261, 262, 263]
	FGF3	Proliferation, survival	STAT3, PI3K/AKT, PLCγ	BC	[264]
	FGFR1	Proliferation, survival, invasion, angiogenesis	MAPK, PI3K/AKT, STAT3, PLCγ	LSCC, BC, OSCC, OC, GBM	[265, 266, 267, 268, 269]
	FGFR2	Proliferation, survival, invasion, migration	PI3K–AKT, MAPK, STAT3, PLCγ, JAK–STAT	GC, TNBC, EC, OC	[270, 271, 272, 273, 274, 275, 276]
	FGFR3	Proliferation, survival, anti‐apoptosis	STAT1, STAT3, PI3K/AKT, RAS/MAPK, PLCγ	Bladder cancer (non‐muscle‐invasive urothelial carcinoma, NMIUC), multiple myeloma with t(4;14) translocation	[277, 278]
	FGFR4	Proliferation, invasion, migration, immune evasion	STAT3, MAPK, PLCγ, JAK/STAT	HCC, GC, OC, NPC, CRC, ARMS	[279, 280, 281, 282, 283]
Gene amplification	*FGF19*	Proliferation, survival, metabolic reprogramming	RAS/MAPK, PI3K/AKT, PLCγ, JAK/STAT	BC, LC, CRC, NPC, HNSCC	[284, 285, 286, 287, 288]
	*FGFR1*	Proliferation, survival, invasion	MAPK, PI3K/AKT, STAT3	LSCC, BC, OSCC, OC, GBM	[265, 266, 267, 268, 269]
	*FGFR2*	Proliferation, survival	PI3K/AKT, MAPK	GC	[270, 271]
Gene mutation	*FGFR1*–*TACC1*	Proliferation, invasion, survival	MAPK, PI3K/AKT, PLCγ, JAK/STAT	BC, NSCLC	[206]
	*FGFR3* (S249C/Y375C/R248C)	Tumor initiation, proliferation	RAS/MAPK, STAT3	BC, CC (S249C)	[289, 290, 291]
	*FGFR3* (K650E)	Proliferation, survival	STAT1, PI3K/AKT	MM	[292]
	*FGFR2* (S252W/P253R)	Proliferation, survival	PI3K/AKT, MAPK	EC	[275, 293]
	*FGFR1* (V561M)	Proliferation, survival	MAPK, PI3K/AKT	LSCC	[294]
	*FGFR1* (K656E)	Tumor initiation, proliferation	PI3K/AKT/mTOR, RAS/MAPK	Glioneuronal tumor	[295]
	*FGFR4* (N535K/V550E)	Proliferation, invasion, migration	STAT3, PI3K/mTOR	ARMS	[283, 296]
	*FGFR4* (Y367C)	Proliferation	MAPK	BC	[296]
	*FGFR4* Gly388Arg (G388R, SNP)	Proliferation, invasion, migration	PI3K/AKT, MAPK, PLCγ	BC, PCa	[297, 298]
	*FGFR2*‐BICC1/CCDC6/AHCYL1/TACC3	Metastasis, invasion, survival	MAPK, PI3K/AKT, STAT	ICC	[299, 300, 301]
	*FGFR1* (N546K)	Tumor initiation, proliferation	MAPK	GBM	[302]
Gene fusion	*FGFR1*–*TACC1*	Proliferation, invasion, survival	MAPK, PI3K/AKT, PLCγ, JAK/STAT	BC, NSCLC	[206]
	*FGFR3*–*TACC3*	Mitochondrial metabolic reprogramming, proliferation	MAPK, PI3K/AKT, PLCγ, STAT	LUSC, HNSCC, NPC	[303, 304, 305, 306, 307]
Aberrant Klotho co‐receptor	KLB overexpression	Proliferation, metastasis, metabolic reprogramming	PI3K/AKT/mTOR	HCC	[308]
	KLB downregulation	Tumor initiation, immune evasion	—	EC, PCa, UDC	[263, 309, 310]

Abbreviations: ARMS, alveolar rhabdomyosarcoma; BC, breast cancer; CC, cervical cancer; CRC, colorectal cancer; EC, endometrial cancer; GBM, glioblastoma; GC, gastric cancer; HCC, hepatocellular carcinoma; ICC, intrahepatic cholangiocarcinoma; IDC, invasive ductal carcinoma; JAK/STAT, Janus kinase/signal transducer and activator of transcription; LC, lung cancer; LSCC, lung squamous cell carcinoma; MM, multiple myeloma; NPC, nasopharyngeal carcinoma; NSCLC, non‐small cell lung cancer; OC, ovarian cancer; OSCC, oral squamous cell carcinoma; PCa, prostate cancer; PCa, prostate cancer; TNBC, triple‐negative breast cancer.

Aberrantly high expression of FGF dominantly drives tumor progression through autocrine and paracrine mechanisms [71]. FGF2, a key regulator of angiogenesis and EMT, exhibits high expression levels that are closely associated with poor prognoses of various solid tumors. For instance, in non‐small cell lung cancer (NSCLC), FGF2 overexpression engages FGFR1/2, with consequent activation of the downstream RAS–RAF–MEK–ERK signaling cascade. This signaling axis stimulates vascular endothelial cell proliferation and concurrently potentiates tumor cell invasion and metastasis [64]. Overexpression of FGF8 plays a significant oncogenic role in multiple types of cancer, including oral squamous cell carcinoma (OSCC) [257], HCC [258], alveolar rhabdomyosarcoma (ARMS) [259], breast cancer [260], prostate cancer [255], and colorectal cancer (CRC) [256]. For example, in prostate cancer, FGF8 activates the MAPK and PI3K–AKT pathways via FGFR1/2, thus propeling malignant cell progression [255]. Elevated FGF8 expression, in addition, drives CRC cell invasion and metastasis through EMT induction and activation of associated signaling cascades [256]. FGF19, a member of the endocrine FGF family, is encoded by a gene located at chromosomal region 11q13.3, which is frequently amplified in multiple cancers. This amplification leads to the overexpression of FGF19 protein and serves as a key driver of tumorigenesis [311]. Within HCC, FGF19 overexpression engages the FGFR4/β‐Klotho complex and activates the downstream RAS–MAPK and PI3K–AKT–mTOR pathways, with the consequence of driving hepatocyte malignant proliferation and suppressing apoptotic cell death [298, 312]. Additionally, FGF19 enhances the antioxidant defense and ER stress tolerance in HCC cells by activating the GSK3β/Nrf2 pathway [261]. And through the FGFR4/SOCE/NFATc2 signaling pathway, FGF19 promotes the self‐renewal of liver cancer stem cells (CSCs) [262]. Beyond HCC, *FGF19* amplification and overexpression have been documented in a range of additional malignancies, including breast cancer [284], lung cancer [285], CRC [286], NPC [287], and head and neck squamous cell carcinoma (HNSCC) [288]. Elevated expression of additional FGF ligands, including FGF3, FGF4, and FGF9, has likewise been reported in breast cancer, gastric cancer, and other cancer types. For example, FGF3 overexpression promotes proliferation and EMT by binding to FGFR1 and activating the STAT3 pathway in breast cancer cells [264]. In TNBC, FGF4 overexpression drives TAMs toward M2 phenotype through activation of IL6/STAT3 signal axis, fostering immunosuppressive microenvironment that favors tumor progression and immune evasion [195].

Overexpression of FGFR receptors also constitutes a major cause of aberrant activation of FGF/FGFR signaling. *FGFR1* amplification has been observed in multiple malignancies, including OSCC [267], ovarian cancer [268], glioblastoma [269], lung squamous cell carcinoma (LSCC), and breast cancer. For instance, in LSCC, *FGFR1* amplification gives rise to protein overexpression and drives malignant progression via an FGF2–FGFR1 autocrine loop. This autocrine circuit sustains constitutive activation of downstream proliferative and survival signals [265]. Moreover, *FGFR1* amplification occurs predominantly in hormone receptor‐positive subtypes of breast cancer and is associated with resistance to endocrine therapy [266]. *FGFR2* gene amplification in gastric cancer results in elevated FGFR2 protein expression, a finding that correlates closely with unfavorable prognosis and lymph node metastasis [270, 271]. Among the various FGFR2 isoforms, FGFR2b is specifically overexpressed in gastric and gastroesophageal junction adenocarcinoma, where it promotes tumor cell proliferation and survival by activating the PI3K–AKT and MAPK signaling pathways [272, 273]. In breast cancer, FGFR2 overexpression has likewise been observed in certain TNBC subtypes, where it suppresses BRCA1 expression via activation of the STAT3 signaling pathway. This action drives TNBC tumorigenesis and fuels malignant progression [274, 313]. In CRC, FGFR2 overexpression can upregulate plasminogen activator inhibitor‐1 (PAI‐1) through activation of JAK2/STAT3 signaling axis. This upregulation drives TAM polarization toward the M2 phenotype, with the effect of establishing an immunosuppressive TME [254]. In addition, FGFR2 overexpression has been documented in various gynecological malignancies, including endometrial cancer (EC) [275] and ovarian cancer [276]. High expression of FGFR3 is relatively common in bladder cancer, particularly in non‐muscle‐invasive urothelial carcinoma. In certain cases, FGFR3 overexpression promotes tumor cell survival and proliferation via activating the STAT1 and STAT3 pathways [277, 278]. Moreover, FGFR3 overexpression is also observed in multiple myeloma, especially in cases harboring the t(4;14) translocation [277]. In addition, overexpression of FGFR4 plays an oncogenic role in various solid tumors. Within HCC, FGFR4 overexpression is associated with tumor invasiveness and poor prognosis, and binding of FGF19 to FGFR4 activates the STAT3 and MAPK pathways, which propels tumor progression [279]. In ovarian cancer, elevated FGFR4 expression is associated with advanced‐stage high‐grade carcinoma. Through MEK–ERK–RSK pathway activation, FGFR4 upregulates the anti‐apoptotic protein Bcl‐xL, thus conferring resistance to paclitaxel. Furthermore, stabilization of FGFR4 protein by SORL1 promotes carboplatin resistance, and targeting FGFR4 has been shown to enhance chemosensitivity [279, 281]. By activating the Wnt/β‐catenin pathway, FGFR4 overexpression facilitates EMT and invasion in CRC [282]. Besides, highly expressed FGFR4 has been observed in NPC tissues and cell lines and correlated with clinical stages and prognosis in NPC patients. In vitro experiments, FGFR4 induces cell proliferation and migration by regulating EMT markers [314]. Additionally, high FGFR4 expression is closely associated with advanced‐stage and poor survival in patients with rhabdomyosarcoma (RMS), and *FGFR4*‐activating mutations promote metastasis in mouse model [283].

Activating mutations within the *FGFR* family, including point mutations and gene fusions, represent key oncogenic drivers across various tumor types [299]. For instance, activating mutations in *FGFR3* (e.g., S249C, Y375C, R248C) are most frequently observed in bladder cancer, and these alterations induce constitutive FGFR3 dimerization, with subsequent activation of the RAS–MAPK and STAT3 axes that drives tumorigenesis. In addition, FGFR3 establishes a positive feedback loop with MYC, conferring an oncogenic dependence [290, 315]. *FGFR3* mutation, including S249C, is detected in cervical cancer [291], whereas the K650E alteration has been identified in multiple myeloma [292]. Additionally, point mutations in *FGFR2* (e.g., S252W, P253R) are highly enriched in EC [275, 293, 316]. *FGFR1* point mutations (V561M) are found in LSCC [294], and *FGFR1* (K656E) mutations are mainly observed in glioblastoma [295]. Besides, *FGFR4* mutations (e.g., N535K, V550E) are common in RMS [283, 296], and *FGFR4* (Y367C) mutation is mainly found in the breast cancer cell line MDA‐MB‐453 [296]. Furthermore, the common *FGFR4* Gly388Arg (G388R) polymorphism (SNP) is associated with poor prognosis in multiple types of cancer, including breast cancer [297] and prostate cancer [298].

FGFR gene fusions constitute another important class of oncogenic events. *FGFR2* gene fusions are characteristic driver alterations in intrahepatic cholangiocarcinoma, with common fusion partners, including *FGFR2–BICC1*, *FGFR2–CCDC6*, *FGFR2–AHCYL1*, and *FGFR2–TACC3*. These fusion proteins drive ligand‐independent and constitutive activation of the FGFR2 kinase domain. The resultant signaling, through activation of the MAPK, PI3K–AKT, and STAT pathways, promotes malignant transformation of cholangiocytes [300, 301, 317]. *FGFR3–TACC3* gene fusions are most prevalent in LUSC [303], IDH‐wild‐type glioma [304], HNSCC [305], and NPC [306]. Notably, this fusion protein can also aberrantly localize to mitochondria, where it induces metabolic reprogramming in tumor cells [307]. Other fusions, such as *FGFR1–TACC1* fusions, have been reported in glioblastoma (including GBM) and specific mesenchymal tissue tumors, including spinal cord glioma and uterine sarcoma [318, 319].

The Klotho protein family exhibits aberrant expression that modulates FGF/FGFR signaling and systemic homeostasis, thereby contributing to the pathogenesis and progression of various tumors [320]. β‐Klotho (KLB) is an essential co‐factor for FGF19 and FGF21 signaling and is predominantly expressed in the liver [321]. KLB is implicated in tumorigenesis, with its expression levels and those of its mutants varying across different cancer types. Elevated KLB expression is significantly associated with poor prognosis of HCC patients [308]. Mechanistically, upregulated KLB promotes the proliferation and migration of HCC cells and enhances FGFR4‐mediated downstream signaling by activating the PI3K/Akt/mTOR pathway. Consequently, silencing of KLB exerts an anti‐proliferative effect in HCC, suggesting that KLB may represent a novel therapeutic target for HCC [308]. However, conflicting evidence also suggests that KLB‐FGFR4 co‐expression induces apoptosis via modulation of the Akt/mTOR pathway, with the consequence of suppressing HCC development [322]. The *KLB* Arg728 allele mutation compromises KLB protein stability and impairs its interaction with FGF19. These effects attenuate downstream signaling and upregulate CYP7A1 expression, collectively predisposing to nonalcoholic fatty liver disease (NAFLD) and cancer [323, 324]. In NSCLC, KLB overexpression alters the cell cycle and inhibits the ERK1/2, AKT, and STAT3 signaling pathways, underling its potential antitumor activity. Accordingly, the serum KLB level may serve as a clinically applicable biomarker for NSCLC diagnosis [325]. In addition, KLB is downregulated in EC [263], prostate cancer [309], and invasive ductal carcinoma of the breast [310], and its overexpression inhibits tumorigenesis. Collectively, these findings underscore that the expression levels and functional mechanisms of KLB vary markedly across different tumors, highlighting its non‐negligible role in oncogenesis.

### 4.2 Attenuated FGF/FGFR Signaling

Conversely, attenuated FGF/FGFR signaling, caused by reduced *FGF* expression, loss of FGFR function, resistance to signaling pathways, or failure of endogenous protective mechanisms, can also contribute to the pathogenesis of various diseases, including metabolic disorders, inflammatory diseases, neurological diseases, and developmental abnormalities.

#### 4.2.1 Metabolic Diseases

Metabolic syndrome is a clustering of metabolic risk factors, including obesity, hypertension, hyperglycemia, and dyslipidemia, and is often accompanied by complications such as Type 2 diabetes and NAFLD/NASH [326]. FGF21 is an endocrine hormone primarily secreted by the liver. Through engagement with the FGFR1c/β‐Klotho complex, FGF21 activates the downstream AMPK signaling cascade. This activation upregulates PPARα and PGC‐1α, with consequent stimulation of FAO and ketogenesis. Moreover, FGF21 also enhances energy expenditure and facilitates GLUT4 translocation, displaying the capability of reducing plasma glucose and triglycerides, as well as improving insulin sensitivity [327]. However, in patients with obesity and metabolic syndrome, although circulating FGF21 levels are significantly elevated in a compensatory manner, its metabolic regulatory function is impaired, a phenomenon known as FGF21 resistance [328]. Exogenous administration of recombinant FGF21 or its long‐acting analogs has been demonstrated to elicit significant improvements in metabolic parameters, as evidenced by both preclinical animal studies and clinical trials [17, 329]. FGF21 also exhibits antioxidant properties. Mechanistically, it activates the Nrf2 signaling axis, potentiates antioxidant enzyme activity, and attenuates oxidative stress‐induced cellular damage [330]. Collectively, these findings establish FGF21 not only as a biomarker for metabolic syndrome and related diseases, but also as an important therapeutic target.

FGF19 serves as a key signaling molecule in the gut–liver axis [331]. Following meal, elevated intestinal BA levels activate the farnesoid X receptor (FXR), which induces the secretion of FGF19 from intestinal epithelial cells. Upon reaching the liver, FGF19 engages the FGFR4/β‐Klotho receptor complex on hepatocytes and subsequently activates downstream signaling pathways and represses *CYP7A1* expression. This negative feedback mechanism prevents excessive BA synthesis and hepatotoxicity, preserving BA metabolic homeostasis [332]. However, once FGF19 signaling is disrupted, *CYP7A1* becomes overexpressed, leading to BA accumulation in the liver. These accumulated BAs trigger mitochondrial dysfunction and ROS overproduction, which, in turn, inflict damage on hepatocytes [333, 334]. Moreover, chronic BA accumulation further triggers liver inflammation, activation of HSCs, and collagen deposition, ultimately progressing to liver fibrosis, cirrhosis, and even HCC [332, 335]. Insufficient FGF19 signaling, beyond its effects on BA synthesis, may compromise biliary epithelial cell integrity, further worsening BA accumulation [336]. Under conditions of BA accumulation, FGF19 upregulates the expression of multidrug resistance‐associated proteins (MRP)‐3/4 via activation of the ERK pathway, thereby mitigating hepatocyte damage [337]. The therapeutic promise of these insights has been borne out in both animal models and human trials, where FGF19 analogs have shown efficacy in cholestatic conditions like primary biliary cholangitis (PBC) or primary sclerosing cholangitis (PSC), as well as metabolic disorders including NASH. The underlying mechanism centers on reduced BA production and attenuated liver fibrosis [332].

#### 4.2.2 Inflammatory Diseases

Sepsis, defined as life‐threatening organ dysfunction caused by a dysregulated host response to infection, and systemic inflammatory response syndrome (SIRS), an excessive systemic inflammatory response triggered by either infectious or non‐infectious stimuli, represent two critical clinical conditions [338]. In patients with sepsis and experimental animal models, plasma FGF21 levels rise sharply during the early stage of the disease, reflecting an adaptive stress response [339]. Studies have demonstrated that exogenous administration of FGF21 significantly improves survival rates and alleviates multiple organ dysfunction in septic mice [340]. Mechanistically, by restoring autophagic flux in macrophages and promoting p62‐mediated autophagic degradation of hypoxia‐inducible factor 1 alpha (HIF‐1α), FGF21 curbs pro‐inflammatory macrophage activation and dampens the inflammatory storm [341]. Similar to FGF21, FGF1 also exhibits anti‐inflammatory and organ‐protective properties. In septic mice, FGF1 suppresses inflammatory cytokine release through inhibition of IL‐6/STAT3 pathway [342]. Beyond its direct anti‐inflammatory action, FGF1 preserves endothelial barrier integrity, suppresses apoptosis, and stimulates angiogenesis, ultimately mitigating liver injury and coagulation dysfunction [343]. Under conditions of excessive inflammation such as sepsis and IBD, endogenous protective FGF/FGFR signaling may be insufficient to counteract the intense inflammatory response, suggesting that exogenous supplementation with FGF21 or FGF1 holds therapeutic potential.

#### 4.2.3 Nervous System Diseases

Epilepsy is a chronic neurological disorder characterized by recurrent and transient central nervous system dysfunctions resulting from highly synchronized abnormal neuronal discharges in the brain [344]. FGF/FGFR signaling plays a regulatory role in both epileptogenesis and synaptic plasticity. FGF22 and FGF7 exert opposing effects on synapse formation: FGF22 tends to promote excitatory synapse formation, whereas FGF7 tends to facilitate the formation of inhibitory synapses [155]. In *Fgf22* knockout mice, the aggregation of excitatory synaptic vesicles in the CA3 region of the hippocampus is reduced, and the miniature excitatory postsynaptic currents (mEPSCs) are diminished, leading to relatively enhanced synaptic inhibition. Consequently, these mice exhibit reduced susceptibility to electrically induced epileptic seizures [345], suggesting that the absence or downregulation of FGF22 signaling exerts an antiepileptic effect by reducing excitatory synapses. Conversely, insufficient or absent FGF7 signaling leads to reduced levels of the VGAT protein at the presynaptic terminals of GABAergic neurons projecting to the CA3 region, a decrease in the frequency of mIPSCs, and a shift in the excitation/inhibition (E/I) balance toward excitability, ultimately increasing susceptibility to seizures [155, 345].

Neurodegenerative diseases, including Alzheimer's disease and Parkinson's disease, are characterized by the progressive loss and dysfunction of specific neuronal populations, accompanied by pathological processes such as protein misfolding and aggregation, mitochondrial dysfunction, oxidative stress, and neuroinflammation [346]. The FGF family has attracted considerable attention for its neurotrophic, neuroprotective, neurogenic, and anti‐inflammatory properties in neurodegenerative disorders [347]. In an in vitro model of embryonic midbrain dopaminergic neurons, FGF2 exerts significant trophic and protective effects on dopaminergic neurons. This action is achieved through stimulation of astrocytes and synergistic cooperation with factors such as TGF‐β [88]. Additionally, in an animal model of Alzheimer's disease, FGF21 enhances antioxidant defense by activating the Nrf2 pathway, thereby reducing oxidative damage to amyloid‐beta (Aβ) and tau proteins. Simultaneously, FGF21 also inhibits the NF‐κB pathway, leading to decreased release of pro‐inflammatory factors, reduced levels of Aβ and phosphorylated tau, and improved cognitive function [348]. Nevertheless, most of these studies remain at the preclinical stage, and their translation into clinical application faces numerous challenges.

Sensory ataxia refers to a condition marked by impaired proprioception that gives rise to pronounced ataxia when visual input is compromised, for instance, with eyes closed. Genetic factors are among the primary etiologies of this disease [349, 350, 351]. Studies have demonstrated that in mice lacking the *FGF14* gene, voltage‐gated sodium channels in Purkinje neurons and granule cells exhibit dysfunction, and the animals display significant ataxia and paroxysmal dyskinesia [352]. Loss of FGF14 leads to reduced Nav channel expression and aberrant channel kinetics, which subsequently impairs the initiation and propagation of action potentials [353, 354]. In humans, mutations in *FGF14* are associated with autosomal dominant cerebellar ataxia. These mutations prevent the FGF14 protein from normally binding to the Nav channel alpha subunit [355]. Furthermore, mutant FGF14 interferes with wild‐type FGF14 function through a dominant‐negative mechanism, giving rise to clinical features including progressive ataxia, paroxysmal dyskinesia, and cognitive decline [356].

#### 4.2.4 Chronic Wound Healing Disorder

Chronic wounds are defined as those that fail to restore normal anatomical structure and functional integrity, frequently marked by a healing process that stalls in the inflammatory phase, driven by factors such as persistent inflammation, impaired angiogenesis, or cellular senescence [357]. The FGF family plays a crucial role in orchestrating the wound healing process. During the early stages of normal wound healing, FGF2, primarily released by platelets, macrophages, and injured endothelial cells, significantly promotes the proliferation and migration of fibroblasts, endothelial cells, and keratinocytes [75]. Moreover, by inducing EMT, FGF2 enables keratinocytes to acquire migratory capacity, which is essential for granulation tissue formation and epithelial regeneration [76, 358, 359]. Dermal fibroblasts secrete FGF7 as a paracrine factor, which drives keratinocyte proliferation via its cognate receptor FGFR2IIIb. During wound re‐epithelialization, FGF7 levels are remarkably upregulated, subsequently enhancing cell migration by increasing the activity of urokinase‐type plasminogen activator (uPA) and reducing apoptosis induced by ROS. Together, these effects of FGF7 facilitate the completion of re‐epithelialization process [96, 97]. However, in chronic wounds, multiple factors collectively contribute to FGF/FGFR signaling dysfunction. Markedly elevated protease activity leads to excessive degradation of growth factors such as FGF2 [360]. A hyperglycemic microenvironment also reduces cellular responsiveness to growth factors [361]. Moreover, a persistent inflammatory milieu inhibits cell proliferation and matrix remodeling [362, 363], leading to pro‐repair signaling pathways, particularly those mediated by FGF2 and FGF7, that are impaired. As a result, wound healing becomes stalled in the inflammatory phase, granulation tissue formation is compromised, and re‐epithelialization remains incomplete [364]. Consequently, local administration of recombinant FGF2 or FGF‐based topical therapies represents an effective strategy for managing chronic wounds.

#### 4.2.5 Excessive Bone Growth

Gain‐of‐function mutations in *FGFR3* cause a spectrum of skeletal dysplasias characterized by disproportionate short stature, including ACH and thanatophoric dysplasia (TD). Conversely, loss‐of‐function mutations in *FGFR3* underlie CATSHL syndrome, defined by camptodactyly, tall stature, scoliosis, and sensorineural hearing loss, a condition marked by excessive bone growth [365, 366, 367]. To date, several families with CATSHL syndrome have been reported, carrying either heterozygous or homozygous pathogenic variants in *FGFR3*, including p.Arg621His, p.Gly369Cys, and p.Thr546Lys. All identified variants reside within the RTK domain and lead to loss of FGFR3 signaling function. At the mechanistic level, inactivating FGFR3 variants attenuate the activities of STAT and MAPK pathways. Consequently, expression of key cell cycle inhibitors, including p16, p18, and p19, is downregulated, leading to enhanced chondrocyte proliferation. Consistent with this mechanism, *Fgfr3* knockout mice display markedly elongated spines and long bones, accompanied by an expansion of the hypertrophic zone and delayed exit from the proliferative zone in the growth plate [368].

In conclusion, dysregulated FGF/FGFR signaling, whether due to pathological hyperactivation or loss‐of‐function, disrupts systemic homeostasis and contributes to the pathogenesis of diverse diseases. This bidirectional nature of FGF/FGFR signaling underscores the functional pleiotropy and context‐dependent regulatory precision of this signaling network.

## 5 Strategies Targeting FGF/FGFR Signaling

The pivotal role of FGF/FGFR signaling in diverse physiological and pathological processes positions it as a promising therapeutic target for a broad spectrum of diseases, including cancer, metabolic disorders, and developmental abnormalities. To address either pathological hyperactivation or functional deficiency of this pathway, a diverse and multi‐level intervention strategy has been developed. These strategies modulate FGF/FGFR signaling through distinct mechanistic modalities, such as blocking signal transduction, inducing receptor degradation, or employing FGF ligand analogs. Collectively, these strategies have demonstrated substantial efficacy in preclinical models and clinical studies (Table 2).

**TABLE 2:** Therapeutic drugs targeting the fibroblast growth factor (FGF)/FGF receptor (FGFR) signaling pathway.

Therapeutic strategy	Category	Drug	Targets	Disease	Clinical stage	References
Strategies targeting the abnormal activation of FGF/FGFR signaling	Non‐selective TKIs	Erdafitinib	FGFR1–4	Cancers with *FGFR* mutations	Clinically approved	[369]
		Dovitinib (TKI258)	FGFR1–4	Cancers with *FGFR* mutations	Phase II (NCT01741116)	[370]
		Rogaratinib	FGFR1–4	Cancers with *FGFR* mutations	Phase II/III (NCT03410693)	[371]
		ARQ 087 (derazantinib)	FGFR1–4	Cancers with *FGFR* mutations	Phase I/II (NCT01752920)	[372]
		KIN‐3248	FGFR1–4	Cancers with *FGFR* mutations	Phase I (NCT05242822)	[373]
		Futibatinib	FGFR1–4	Cancers with *FGFR* mutations	Clinically approved	[374]
	Selective TKIs	Pemigatinib	FGFR1–3	Cancers with *FGFR* mutations	Clinically approved	[375]
		AZD4547	FGFR1–3	Cancers with *FGFR* mutations	Phase I/II (NCT02824133)	[376]
		Infigratinib	FGFR1–3	Cancers with *FGFR* mutations	Clinically approved	[377]
		BLU9931	FGFR4	Cancers with high FGFR4 expression	Preclinical	[378]
		BLU‐554 (fisogatinib)	FGFR4	HCC	Phase I/II (NCT04194801)	[379]
		H3B‐6527	FGFR4	HCC	Phase I (NCT02834780)	[380]
		Irpagratinib (ABSK‐011)	FGFR4	HCC	Phase II (NCT07010497)	[381]
		RLY‐4008	FGFR2	iCCA	Phase I/II (NCT04526106)	[382]
	Monoclonal antibody	R3Mab	FGFR3	Tumor	Phase I/II (NCT02401542)	[383]
		U3‐1784	FGFR4	HCC	Phase I (NCT02690350)	[384]
		Bemarituzumab (FPA144)	FGFR2b	Tumor	Phase I (NCT05913115)	[385]
	Antibody–drug conjugate	LY3076226	FGFR3	Tumor	Phase I (NCT02529553)	[386]
		Aprutumab ixadotin (BAY 1187982)	FGFR2	Tumor	Phase I (NCT02368951)	[387]
	Ligand trap	FP‐1039 (GSK3052230)	FGF ligands	Tumor	Phase I (NCT01868022)	[388]
	Aptamer	RSc6/RSc37 (RNA aptamers)	FGFR3	Skeletal development regulation	Preclinical	[389]
		RBM‐007 (anti‐FGF2 aptamer)	FGF2	Wet AMD, ACH	Phase II (NCT04640272)	[390]
		TD0 (DNA aptamer)	FGFR1	Stem cell culture, tissue regeneration	Preclinical	[391]
	Cell therapy	FGFR4‐CAR T cells	FGFR4	RMS, HCC, CCA	Preclinical	[357]
		FGFR4/CD276 bispecific CART	FGFR4/CD276 (B7‐H3)	RMS	Preclinical	[392]
	Gene therapy	AAV9FGFR2shRNA	Mutant FGFR2P253R	Apert syndrome	Preclinical	[393]
		CRISPR/Cas9 correction	Mutant FGFR3G374R	Dwarfism	Preclinical	[394]
Strategies targeting the inactivation of FGF/FGFR signaling function	FGF analogs	LY2405319 (LUM‐201)	FGF21	T2DM, obesity, NASH	Preclinical	[329]
		Repifermin (rFGF10)	FGF10	Oral and intestinal mucositis	Phase II (PMID: 11896977)	[395]
		NGM282 (aldafermin)	FGF19	NASH, PBC	Phase II (NCT02443116)	[396]
		Sprifermin (rhFGF18)	FGF18	OA	Phase II (NCT01689337)	[397]
		LY2405319 (LUM‐201)	FGF21	T2DM, obesity, NASH	Preclinical	[329]
		Burosumab	FGF23	XLH, TIO, ARHR1	Phase II (NCT02163577)	[398]
		M70	FGF19 variant	NASH, PBC	Preclinical	[399]
		Efruxifermin (AKR‐001)	FGF21	NASH	Phase II (NCT04767529)	[400]
		Pegbelfermin (BMS‐986036)	FGF21	NASH	Phase II (NCT03486912)	[401]
	Gene therapy	NV1FGF	FGF1	CLI	Phase III (NCT00566657)	[402]
		Ad5FGF‐4	FGF4	IHD	Phase II/III (NCT00346437)	[403]
		AAV FGF18	FGF18	ACH	Preclinical	[404]

Abbreviations: AAV, adeno‐associated virus; ACH, achondroplasia; AMD, age‐related macular degeneration; ARHR1, autosomal recessive hypophosphatemic rickets Type 1; Bi‐CAR‐T, bispecific CAR‐T; CCA, cholangiocarcinoma; CLI, critical limb ischemia; HCC, hepatocellular carcinoma; iCCA, intrahepatic cholangiocarcinoma; IHD, ischemic heart disease; NASH, nonalcoholic steatohepatitis; OA, osteoarthritis; PBC, primary biliary cholangitis; RMS, rhabdomyosarcoma; T2DM, Type 2 diabetes mellitus; TIO, tumor‐induced osteomalacia; TKIs, tyrosine kinase inhibitors; XLH, X‐linked hypophosphatemia.

### 5.1 Strategies Targeting the Abnormal Activation of FGF/FGFR Signaling

Aberrant activation of the FGF/FGFR signaling contributes to the pathogenesis of multiple disorders, including abnormal bone development, metabolic and endocrine diseases, as well as diverse malignancies. Currently, several targeted therapeutic strategies have been developed to inhibit this hyperactivated axis, including small‐molecule tyrosine kinase inhibitors (TKIs), proteolysis‐targeting chimeras (PROTACs), mAbs and antibody‐derived therapeutics, chimeric antigen receptor T‐cell (CAR‐T) therapy, and gene interference approaches.

#### 5.1.1 Small‐Molecule TKIs

Small‐molecule TKIs currently represent the most extensively developed class of FGFR‐targeted agents in clinical practice. These agents competitively inhibit FGFR kinase activity by occupying the ATP‐binding pocket, thereby suppressing downstream signaling cascades [405, 406]. Pan‐FGFR inhibitors exhibit differential inhibitory potency against FGFR1–4 and are indicated for tumors harboring diverse FGFR genomic alterations, including gene amplifications, mutations, and fusions.

Erdafitinib, an oral small‐molecule FGFR TKI, that has been approved for patients with advanced urothelial carcinoma carrying FGFR2 or FGFR3 alterations. In the Phase II RAGNAR trial, erdafitinib achieved an objective response rate of approximately 30% across a spectrum of FGFR‐altered solid tumors [369]. Futibatinib is an irreversible, covalent FGFR inhibitor that achieves durable target suppression through forming a stable bond with a conserved cysteine residue within the FGFR kinase domain. It has been approved for patients with advanced intrahepatic cholangiocarcinoma harboring FGFR2 fusion or rearrangement. Clinical evidence also shows its therapeutic activity in other solid tumors driven by oncogenic FGFR alterations [374, 407, 408]. Pemigatinib is a selective FGFR1‐3 inhibitor that demonstrates clinically meaningful efficacy in patients with advanced cholangiocarcinoma carrying FGFR2 fusions or other rearrangements and has received approval for this indication [375], with its indications gradually expanding to additional FGFR‐altered malignancies, including urothelial carcinoma [409]. AZD4547 is an ATP‐competitive inhibitor that has shown potent activity in preclinical studies [410]. However, its clinical translation has been limited by modest efficacy and tolerability challenges in human trials. It has been evaluated in multiple clinical studies, including in patients with *FGFR2*‐amplified gastric cancer [411], endocrine‐resistant breast cancer [412], and LSCC [413]. Other pan‐FGFR inhibitors under clinical investigation include dovitinib (TKI258) [414], rogaratinib (with patient selection based on FGFR1/3 mRNA expression) [371], infigratinib (BGJ398) [377], ARQ087 [415], and the next‐generation irreversible inhibitor KIN‐3248 [416].

FGFR4 plays a specific oncogenic role in FGF19‐driven HCC [417, 418], whereas pan‐FGFR inhibitors targeting FGFR1–4 frequently induce dose‐limiting toxicities, including hyperphosphatemia. Therefore, the development of highly selective FGFR4 inhibitors has emerged as a major therapeutic priority. BLU9931, an early‐generation, covalent FGFR4 inhibitor, has not advanced to clinical approval but remains a foundational chemical tool that provided critical proof‐of‐concept and high‐resolution structural insights for subsequent FGFR4‐targeted drug design [378]. Fisogatinib (BLU‐554) is an irreversible FGFR4 inhibitor rationally engineered from BLU9931, and its high selectivity for FGFR4 is achieved through covalent engagement of Cys552 within the FGFR4 kinase domain [419]. Preclinical studies demonstrate that BLU‐554 induces potent cytotoxicity and cellular senescence in FGFR4‐activated cell lines [420]. Additionally, H3B‐6527 represents another highly selective FGFR4 inhibitor [421], and it completed a Phase I clinical trial demonstrating favorable tolerability and preliminary antitumor activity in patients with HCC harboring either FGF19 overexpression or activating *FGFR4* mutations [380]. ABSK‐011 (irpagratinib), also an irreversible FGFR4 inhibitor, has progressed to late‐stage clinical trials. Compared with fisogatinib, irpagratinib achieves more sustained target inhibition through its unique covalent bonding mechanism and has shown preliminary efficacy in patients with FGF19‐overexpressing advanced HCC [422].

With the widespread clinical application of FGFR inhibitors, acquired resistance has gradually emerged as a principal limitation to durable therapeutic response. Several resistance mechanisms have been identified to date. One well‐documented category involves secondary kinase domain mutations, such as the *FGFR2* gatekeeper mutation (V565I) and multiple‐site mutations, which either sterically hinder inhibitor binding to the ATP‐binding pocket or stabilize the active kinase conformation, thereby reducing drug affinity [423, 424]. Alternatively, tumor cells may maintain signaling flux through downstream RAS–RAF–MEK–ERK and PI3K–AKT–mTOR pathways via activation of bypass signaling routes, including EGFR, HER2/HER3, MET, and STAT3. This alternative signaling enables tumor cells to evade FGFR inhibitor‐mediated suppression [294, 425, 426]. Additionally, prolonged exposure to FGFR inhibitors can also promote EMT, driving some tumor cells to acquire resistance [427]. To address these challenges, the next generation of FGFR inhibitors have been rationally designed to retain potency against a broader spectrum of genetic aberrations and clinically observed resistance variants. RLY‐4008 (lirafugratinib) is a first‐in‐class, highly selective, irreversible FGFR2 inhibitor approved for patients with previously treated, locally advanced or metastatic cholangiocarcinoma harboring *FGFR2* fusions, rearrangements, or activating mutations. It maintains robust activity against gatekeeper and other resistance‐conferring *FGFR2* mutations, including V565I, and demonstrates clinically meaningful efficacy in biomarker‐selected patients, with a manageable safety profile dominated by on‐target hyperphosphatemia [428]. KIN‐3248 is another irreversible pan‐FGFR inhibitor engineered to overcome kinase domain mutation‐mediated resistance across FGFR2 and FGFR3, and preclinical and Phase I data support its activity in resistant models and early clinical signals of antitumor efficacy, though its development was terminated early for commercial reasons [416]. For context‐specific resistance mechanisms, such as STAT3 activation and EMT driven by the *FGFR1* V561M mutation, rational combination strategies (e.g., FGFR plus STAT3 or MEK inhibition) are under active clinical investigation as an effective strategy to overcome resistance [294].

#### 5.1.2 Protein Degradation‐Targeting Chimeras

PROTACs represent an innovative therapeutic modality that induces selective degradation of target proteins through recruitment to E3 ubiquitin ligases, leading to polyubiquitination and subsequent proteasomal destruction [429, 430]. At the core of this strategy are heterobifunctional molecules, comprising a target‐binding ligand, a linker, and an E3 ligase–binding moiety, that physically bridge the target protein and the ubiquitination machinery. To date, PROTACs directed against FGFR1 [431, 432], FGFR2 [433, 434], and FGFR3 [435] have demonstrated robust antitumor activity in preclinical studies by achieving sustained target depletion, offering a mechanistically distinct approach to circumvent resistance mechanisms associated with conventional FGFR‐TKIs. Furthermore, emerging innovations include photocontrolled PROTACs, engineered to undergo structural activation only upon exposure to specific wavelengths of light (e.g., ultraviolet or near‐infrared), enabling precise spatiotemporal control of protein degradation. This strategy significantly reduces off‐target effects and enhances on‐target specificity, thereby improving the therapeutic index [436].

#### 5.1.3 mAbs and Their Derivatives

mAbs exert their therapeutic effects through a mechanism fundamentally distinct from that of small‐molecule TKIs. These agents bind with high affinity and specificity to the extracellular domain of FGFR receptors or to FGF ligands, thereby sterically inhibiting ligand–receptor engagement and downstream signal initiation [55, 437]. Unlike TKIs, mAbs do not interfere with intracellular kinase activity; instead, they act extracellularly to prevent pathway activation at its origin. This mechanism confers several pharmacological advantages, including prolonged serum half‐life, minimal off‐target kinase‐related toxicities (e.g., hyperphosphatemia, retinal pigment epithelial detachment), and reduced potential for on‐target resistance mutations in the kinase domain. Consequently, they represent a clinically viable therapeutic strategy for malignancies and skeletal disorders driven by dysregulated FGF/FGFR signaling.

Currently, mAb therapies targeting FGFR receptors have demonstrated substantial antitumor potential in both preclinical and clinical trials. Bemarituzumab is a humanized immunoglobulin G 1 (IgG1) mAb that selectively targets the FGFR2b isoform, and its Fc segment has been modified via glycoengineering to enhance antibody‐dependent cell‐mediated cytotoxicity (ADCC). In the first‐line treatment of patients with HER2‐negative advanced gastric or gastroesophageal junction adenocarcinoma exhibiting FGFR2b protein overexpression, bemarituzumab significantly improved progression‐free survival and overall survival when combined with the mFOLFOX6 chemotherapy regimen [385, 438]. This clinical success not only established FGFR2b overexpression as a clinically validated predictive biomarker but also accelerated the development of AI‐based pathological screening technologies [439, 440]. Furthermore, U3‐1784 is a humanized anti‐FGFR4 mAb engineered with reduced binding affinity to preserve BA homeostasis and mitigate hepatotoxic risk, a key safety consideration given FGFR4's physiological role in BA regulation [441]. Additional FGFR‐targeted antibodies under investigation include antagonistic anti‐FGFR1 antibodies [442], selective anti‐FGFR2c antibodies [443], and anti‐FGFR3 antibodies developed either as standalone therapeutics or as payloads for antibody–drug conjugates (ADCs) [444]. Notably, the R3Mab antibody directed against FGFR3 blocks ligand binding and induces ADCC, demonstrating potent antitumor activity against bladder cancer and multiple myeloma [277].

An alternative approach to inhibit aberrant activation of the FGF/FGFR signaling is ligand neutralization, specifically targeting pathologically elevated circulating FGF ligands. For example, although neutralizing antibodies targeting FGF19 can suppress tumor growth, they are often associated with severe metabolic disorders, such as BA synthesis imbalance, intestine malabsorption, and diarrhea [445]. To mitigate these on‐target metabolic adverse effects while preserving the physiological metabolic function of FGF19, epitope‐selective antibodies have been engineered to bind the N‐terminal domain of FGF19. Such antibodies potently suppress HCC proliferation without perturbing systemic BA homeostasis, thereby achieving a favorable therapeutic index [446].

Ligand traps represent a decoy‐based therapeutic strategy that sequesters extracellular FGF ligands to prevent receptor activation. These engineered proteins typically comprise the soluble extracellular ligand‐binding domain of an FGFR isoform fused to a human IgG1 Fc domain, conferring high avidity for cognate ligands and extending serum half‐life via FcRn‐mediated recycling [447]. By competitively binding free ligands in circulation, ligand traps sterically hinder formation of the functional FGF–HSPG–FGFR ternary complex, thereby abrogating downstream signaling. FP‐1039 (GSK3052230) is a clinical‐stage ligand trap consisting of the FGFR1c extracellular domain fused to an IgG1 Fc fragment; it potently neutralizes multiple FGFR1‐preferring ligands, including FGF2, FGF4, and FGF8, with high affinity. FP‐1039 has demonstrated antitumor activity in malignant pleural mesothelioma (MPM), both as monotherapy and in combination with pemetrexed–cisplatin chemotherapy, and has advanced to Phase II clinical evaluation [448, 449]. Complementary to ligand traps, small‐molecule inhibitors designed to mimic the antiangiogenic domain of thrombospondin‐1 (TSP‐1) have been developed to target the heparin‐binding domain of FGF2. By occupying this site, these compounds disrupt FGF2 dimerization, impair HSPG co‐receptor engagement, and inhibit assembly of the FGF2–HSPG–FGFR1 signaling complex, resulting in potent suppression of FGF2‐driven angiogenesis [450, 451].

At the level of precise killing, ADCs combine the targeting ability of antibodies with highly effective cytotoxic payloads, enabling the specific eradication of tumor cells expressing the relevant receptors. Aprutumab ixadotin (BAY 1187982), an ADC targeting FGFR2, has completed its first‐in‐human Phase I study in patients with advanced solid tumors [387]. LY3076226, an FGFR3‐targeting ADC, has entered early‐stage clinical investigation and demonstrates robust pro‐apoptotic activity in inducing apoptosis in FGFR3‐driven malignancies [444]. Furthermore, ADCs targeting FGFR4 are also under investigation for indications such as RMS and HCC, where FGFR4 dysregulation, driven by FGF19 overexpression or activating mutations, presents a compelling therapeutic vulnerability. Although most FGFR4‐targeted ADC programs remain in preclinical or early clinical stage, their strategy of specifically delivering cytotoxic agents offers a promising avenue to address the limited efficacy and on‐target toxicities associated with conventional FGFR4 inhibitors [452].

In diseases such as XLH and TIO, pathologically elevated circulating FGF23 is the central mediator of renal phosphate wasting and consequent hypophosphatemia. To counteract this mechanism, fully human mAbs targeting FGF23 have been developed to selectively neutralize excess FGF23 in circulation. Burosumab, a human IgG1 mAb, binds the FGF23 N‐terminal domain, preventing its interaction with the FGFR–α‐Klotho receptor complex. It consistently elevates serum phosphate concentrations, improves rickets/osteomalacia severity, and enhances functional outcomes, including mobility, growth velocity in children, and pain scores, in both pediatric and adult XLH patients [453, 454]. On the basis of robust clinical evidence, burosumab has received approval for XLH and, subsequently, for TIO [455]. Complementary to antibody‐based neutralization, Fc fusion proteins comprising the C‐terminal fragment of FGF23 (e.g., FP‐1022) have been engineered as decoy receptors. These agents competitively inhibit FGF23 binding to FGFR–α‐Klotho without globally suppressing FGF23 signaling, thereby preserving its physiological roles in iron metabolism and cardiac function [456]. Concurrently, small‐molecule inhibitors of FGF23 signaling axis are emerging from computational screening and structural simulation efforts. These compounds provide new research tools and potential therapeutic options for pharmacological modulation of the FGF23 pathway [457, 458].

#### 5.1.4 CAR‐T Cell Therapy

CAR‐T therapy involves the ex vivo genetic engineering of autologous T lymphocytes to express surface receptors that confer high‐affinity, antigen‐specific recognition of tumor‐associated surface markers [357]. FGFR4, owing to its relatively restricted and elevated expression in certain solid tumors, has emerged as an attractive target for CAR‐T therapies. Preclinical studies demonstrate that FGFR4‐targeting CAR‐T cells mediate robust cytolytic activity against FGFR4‐positive tumor cells in vitro and induce potent antitumor efficacy in immunocompetent and xenograft RMS models, significantly suppressing tumor growth and promoting near‐complete eradication of established lesions [357, 459].

To mitigate two principal limitations of CAR‐T therapy in solid tumors, antigen heterogeneity‐driven immune escape and on‐target, off‐tumor toxicity, researchers have developed dual‐targeting CAR‐T constructs and inducible safety switch systems. For example, dual‐target CAR‐T cells that recognize both FGFR4 and CD276 (B7‐H3) have been engineered to broaden tumor antigen coverage and reduce the likelihood of immune escape attributable to clonal loss or downregulation of either target antigen [392]. Complementing this approach, inducible caspase‐9 (iCasp9) suicide gene systems have been integrated into FGFR4‐directed CAR‐T cells; administration of the small‐molecule dimerizer drug rimiducid triggers rapid, dose‐dependent apoptosis of >90% of engineered T cells within hours, providing a robust, clinically validated safety mechanism to abrogate severe adverse events [452].

FGFR4‐targeted CAR‐T cells have demonstrated robust antitumor potential in preclinical models of RMS and HCC. However, their clinical translation into solid tumor therapy remains impeded by three well‐characterized barriers, including immunosuppression within the TME, limited cell infiltration efficiency, and the risk of on‐target off‐tumor toxicity. To address these challenges, further optimizing CAR structural design, combining CAR‐T with other immunomodulatory strategies, or implementing precise biomarker screening to identify suitable patient populations will be essential to advance FGFR4‐directed CAR‐T therapy toward clinical utility in solid tumors.

#### 5.1.5 Gene Interference Therapy

Adeno‐associated virus (AAV) vectors constitute a leading platform for in vivo gene delivery [394]. In the context of FGFR‐related monogenic disorders, AAV‐mediated therapeutic strategies have demonstrated robust proof‐of‐concept in multiple animal models. Specifically, for ACH, a skeletal dysplasia caused by gain‐of‐function mutations in *FGFR3*, therapeutic inhibition via a soluble FGFR3 decoy receptor has emerged as a promising approach. Recifercept, a clinically developed recombinant soluble FGFR3 variant, sequesters pathologically elevated FGF ligands, thereby normalizing aberrant downstream signaling and ameliorating key skeletal phenotypes in murine models of ACH [460]. Meanwhile, the CRISPR/Cas9 technology has been successfully employed to correct the *FGFR3* G374R mutation in vivo in murine models of ACH, leading to restoration of the normal skeletal phenotype [461]. In the Apert syndrome model driven by the *FGFR2* S252W mutation, RNA interference (RNAi) has been employed to deliver short hairpin RNA (shRNA) targeting the mutant allele, enabling selective suppression of mutant *FGFR2* expression and alleviating craniosynostosis symptoms in mouse models [462].

RNAi and antisense oligonucleotides (ASO) can suppress pathway activity by silencing the expression of specific *FGFR* or *FGF* gene. Preclinical studies have demonstrated that targeted silencing of the *FGFR4* gene using specific siRNAs attenuates downstream oncogenic signaling, including ERK and STAT3 activation, and induces significant reductions in tumor cell proliferation, invasion, and metabolic capacity [314, 463, 464, 465]. Similarly, siRNA‐driven silencing of specific FGF ligands (e.g., FGF2, FGF11) has provided mechanistic insights into their functional roles in tumor initiation and progression [466, 467]. Advances in nanodelivery systems have substantially addressed the limitations of nucleic acid drugs, particularly with respect to in vivo stability and targeted delivery, thereby substantially enhancing their translational viability [468]. Notably, ASO can also be harnessed to modulate RNA splicing processes and have shown application potential in the study of diseases arising from splicing abnormalities [469].

CRISPR/Cas9 gene editing technology allows knockout or correction of specific genes at the genomic level, establishing it as an indispensable platform for functional dissection of the FGF/FGFR signaling and for identifying potential therapeutic targets [470]. For instance, a CRISPR/Cas9 whole‐genome screening in sorafenib‐resistant HCC revealed FGF21 and the Nrf2 pathway as critical determinants of therapeutic resistance [471]. Conversely, a parallel genome‐wide screen in gastric cancer identified multiple kinase targets that modulate the sensitivity of gastric cancer cells to FGFR inhibitors [472]. Despite its considerable promise in preclinical research, off‐target effects and in vivo delivery efficiency remain major obstacles to clinical translation [473]. At present, application of CRISPR/Cas9 in FGF/FGFR signaling research remains exclusively preclinical; nevertheless, its unparalleled capacity to establish causal genotype provides foundational insights into pathway biology and to inform the rational design of next‐generation targeted therapeutics.

#### 5.1.6 Combined Treatment Strategy

The tumor signaling network exhibits profound complexity and robust adaptive redundancy. Consequently, monotherapeutic inhibition of a single node, such as an individual FGFR or downstream effector, frequently yields transient responses and is commonly associated with the emergence of acquired resistance [474]. In HCC, preclinical evidence demonstrates that pharmacologic or genetic inhibition of the FGF19/FGFR4 signaling reprograms the immunosuppressive TME, providing a strong rationale for combining this pathway inhibition with immune checkpoint inhibitors (ICIs) [475]. Additionally, several combination regimens targeting FGFR signaling alongside immune checkpoint blockade have demonstrated encouraging clinical activity in early‐phase trials. For instance, the combination of futibatinib and pembrolizumab (anti‐PD‐1) exhibited preliminary antitumor efficacy in patients with advanced urothelial carcinoma and esophageal cancer, and the safety profile of this regimen has been generally manageable [422, 476]. Concurrently, a Phase I/II trial evaluating erdafitinib combined with cetrelimab (anti‐PD‐1) is ongoing in patients with advanced solid tumors in Japan [477]. In addition, a Phase I study of a selective FGFR4 inhibitor (EVER4010001) in combination with pembrolizumab has completed dose exploration and shown good tolerability [478]. The combination of multi‐target TKI lenvatinib with ICIs has yielded substantial clinical benefits in HCC and other tumor types [479, 480].

For acquired resistance arising from bypass signaling activation, combination strategies integrating FGFR inhibitors with other targeted agents have emerged as a critical therapeutic paradigm. In HCC models, upregulation of EGFR signaling constitutes a key mechanism of resistance to FGFR4 inhibitors. Concurrent pharmacologic blockade of EGFR using agents such as erlotinib or gefitinib not only re‐sensitizes tumor cells to FGFR4 inhibitors but also significantly enhances their antitumor efficacy [425]. In lung adenocarcinoma, FGFR4 potentiates EGFR‐driven oncogenic signaling, and dual pharmacologic inhibition of FGFR4 and EGFR elicits synergistic antitumor effects in preclinical models, including suppression of tumor growth and induction of apoptosis [426]. In hormone receptor‐positive (HR+)/human epidermal growth factor receptor 2‐negative (HER2−) metastatic breast cancer, the triple combination of the pan‐FGFR inhibitor erdafitinib, the endocrine therapy drug fulvestrant, and the cyclin‐dependent kinase 4/6 (CDK4/6) inhibitor palbociclib has advanced to Phase Ib clinical investigation [481]. In addition, given the role of FGFR signaling in regulating extensive alternative splicing of genes, on this basis, combined inhibition of FGFR and splicing regulatory kinase (e.g., serine/arginine protein kinase, SRPK, or CDC2‐like kinase, CLK) has been shown to synergistically induce tumor cell apoptosis; this strategy has shown therapeutic potential in models such as cholangiocarcinoma [482, 483, 484]. Besides, studies have indicated that combining FGFR pathway inhibitors with conventional chemotherapy agents can simultaneously target the rapidly proliferating cell population and its key dependent pathways, achieving more effective tumor suppression. For example, FP‐1039, an FGF ligand trap, is being evaluated in a Phase II clinical trial (NCT02599754) in combination with pemetrexed and cisplatin for unresectable malignant pleural mesothelioma, and this regimen blocks FGF/FGFR signal to inhibit tumor angiogenesis and cell proliferation [449].

### 5.2 Strategies Targeting the Inactivation of FGF/FGFR Signaling Function

Functional impairment or deficient expression of FGF/FGFR signaling also contributes to a range of pathological conditions, including developmental abnormalities and impaired tissue repair. Current therapeutic strategies aimed at restoring pathway activity primarily encompass FGF analogs, aptamers, and gene delivery approaches.

#### 5.2.1 FGF Analogs

Inactivation of FGF/FGFR signaling contributes to the pathogenesis of multiple diseases, such as metabolic dysregulation and impaired tissue repair. To therapeutically restore pathway function, engineered FGF analogs have been developed as a targeted biologics strategy. These analogs are generated by modifying or directly utilizing FGF ligands, particularly endocrine‐type FGFs, and optimizing their pharmacokinetic properties and target specificity through protein engineering. Clinically, FGF analogs are primarily employed for the treatment of metabolic diseases and the promotion of tissue repair [1, 485].

FGF19 plays a crucial physiological role in BA metabolism and the maintenance of glucose homeostasis. However, its mitogenic activity has been confirmed to be closely associated with the risk of HCC [417, 418]. Consequently, retaining the metabolic functions of FGF19 while attenuating its tumorigenic potential represents a central challenge for its clinical application. Leveraging structural biology studies, researchers have systematically dissected the functional domains of FGF19 involved in metabolic regulation versus cell proliferation and have engineered variants that markedly diminish the activation of tumorigenic signaling pathways while preserving the metabolic effects of FGF19 [399, 486]. Notably, these modified FGF19 chimeric molecules have been shown to promote regeneration of fatty liver tissue without inducing tumor formation [487]. FGF19 analogs developed based on this strategy (e.g., NGM282/aldafermin, a 190‐AA peptide, is engineered through domain modification to reduce oncogenic activity while retaining metabolic function) have advanced into clinical trials, primarily for the treatment of NASH and PBC, demonstrating broad promise in the management of metabolic liver diseases [17].

FGF21 is an important endocrine factor involved in regulation of glucose and lipid metabolism. However, its clinical application as a direct injectable therapeutic is limited by its extremely short half‐life in the body and susceptibility to enzymatic degradation [327, 488]. LY2405319 (LUM‐201), a modified FGF21 analog, significantly improves lipid profiles and increases leptin levels in patients with obese Type 2 diabetes mellitus, although its direct glucose‐lowering effect remains relatively modest [329]. Efruxifermin (AKR‐001), an FGF21‐Fc fusion protein developed by Akero Therapeutics, markedly reduces body weight, enhances glycemic control, and has demonstrated potential in reversing liver fibrosis in patients with NASH through high‐affinity binding to the β‐Klotho receptor [489, 490]. PEGylation represents a well‐established protein engineering strategy to enhance the pharmacokinetic and biophysical properties of therapeutic peptides. This approach involves covalent attachment of polyethylene glycol (PEG) molecules to specific sites on FGF21, with consequent prolongation of circulating half‐life and improvement of solubility and stability [491, 492]. Several PEGylated FGF21 analogs are currently under clinical development. Among them, Pegbelfermin (BMS‐986036) has achieved notable lipid‐lowering effects in Phase II clinical trials for patients with severe hypertriglyceridemia [493]. Another common strategy in PEGylation involves fusing FGF21 with the Fc fragment of IgG. This fusion not only exploits the long half‐life conferred by neonatal Fc receptor (FcRn)‐mediated recycling but may also engage ADCC mechanisms. Importantly, these FGF21 analogs exert their metabolic regulatory effects primarily by activating a receptor complex composed of β‐Klotho and FGFR1c, FGFR2c, or FGFR3c [494]. Additional molecular engineering strategies have been developed on this platform. These include fusion of FGF21 with antibody Fc fragment or covalent linkage to scaffold antibodies, with the goal of achieving sustained delivery by leveraging the extended half‐life of antibodies [495, 496]. For example, LY2405319 has been shown to intervene in the progression of NASH by improving hepatic mitochondrial function [497].

Certain paracrine FGFs, owing to their involvement in cell proliferation and tissue nutritional support, can be harnessed for the repair of various tissue injuries. Repifermin, a recombinant human FGF10, has been evaluated in clinical studies for the alleviation of oral mucositis following hematopoietic stem cell transplantation, with supportive evidence also emerging from pilot studies in active ulcerative colitis [498, 499]. Sprifermin (recombinant human FGF18) has been administered intra‐articularly to patients with knee OA, and follow‐up results demonstrated that it slows the loss of cartilage volume and exerts a sustained effect on symptom improvement [500, 501].

#### 5.2.2 Aptamers

Aptamers are synthetic, single‐stranded DNA or RNA molecules that bind to their target molecules with high specificity. DNA aptamers targeting FGFR1, such as TD0, selected using this platform, function as orthosteric agonists, mimicking natural activity and inducing downstream signaling pathway activation [502]. Additionally, RNA aptamers capable of modulating FGFR3 activity have been isolated [389]. The FGF2‐targeting aptamer RBM‐007 is currently under investigation for the treatment of conditions such as age‐related macular degeneration [503]. Owing to their advantages, including facile synthesis, straightforward chemical modification, and low immunogenicity, aptamers hold significant promise for exploring the biological mechanisms of the FGF/FGFR pathway and for developing novel therapeutic strategies.

#### 5.2.3 Gene Delivery Therapy

Gene therapies enable precise regulation of the FGF/FGFR signaling by directly correcting pathogenic gene defects or delivering functional gene products (e.g., specific FGF ligands or receptor variants). These approaches offer advantages such as single‑administration delivery, long‐term sustained expression, and curative potential. As such, gene therapy is emerging as a complementary therapeutic dimension for FGF/FGFR signaling dysregulation‐associated diseases [504, 505].

For instance, NV1FGF‐1 is a vascular gene therapy approach based on the pCOR plasmid vector, administered via a single intramuscular injection. In the field of vascular regeneration, NV1FGF, which delivers the *FGF1* gene, has been reported to promote angiogenesis and reduce amputation rates in patients with severe limb ischemia [402, 506]. Clinical trials for intermittent claudication and critical limb ischemia have shown that NV1FGF has the potential to lower amputation risks [506]. In another clinical trial using Ad5FGF‐4, a single delivery of the adenovirus vector to cardiac tissue successfully improved myocardial perfusion and significantly reduced the frequency of angina attacks, demonstrating its therapeutic potential in treating ischemic heart disease [507, 508]. In an acute lung injury model, AAV‑mediated overexpression of the *FGF18* gene reduced pulmonary vascular permeability and inhibited NF‐κB pathway activation; the inflammatory response was alleviated as a consequence [509]. In the EC cell line MFE‐280, AAV9‐*FGFR2*‐shRNA significantly suppresses the soft‐agar colony formation, an effect that reflects its antitumor effect [510].

In conclusion, for diseases driven by dysregulated FGF/FGFR signaling, targeted modulation of this pathway through ligand–receptor binding interference or receptor kinase activity inhibition has emerged as a highly effective therapeutic strategy. With increasing knowledge of the protein structures of FGF/FGFR signaling components, an increasing number of specific FGFR inhibitors and FGF analogs are advancing into clinical trials. Nevertheless, several critical challenges remain. (1) The potential oncogenic effects of FGF analogs warrant considerable attention. For instance, the FGF19 analog aldafermin has not entirely eliminated its pro‐tumorigenic activity and exhibits a marked synergistic effect with *MYC*, an oncogene commonly implicated in HCC [9]. Thus, balancing the preservation of metabolic benefits with the maximization of safety constitutes a core challenge for the future clinical translation of FGF analogs. (2) Blockade of FGF/FGFR signaling in tumors may provoke bypass signaling, leading to acquired resistance. For example, following prolonged exposure to FGFR inhibitors, some tumor cells acquire resistance through EMT, accompanied by downregulation of *FGFR* expression and activation of the TGF‐β pathway [477]. Consequently, combined inhibition of FGFR and other key signaling pathways represents a critical strategy to overcome resistance. (3) Precise identification of “beneficiary populations” that are dependent on this pathway is pivotal for the successful implementation of FGF/FGFR targeted therapies. However, standardized clinical detection protocols for FGF/FGFR alterations, such as assays for *FGFR* gene mutations, amplifications, or fusions, have yet to be established. (4) Given the pleiotropic nature of FGF/FGFR signaling, FGFR inhibitors frequently elicit multiple adverse effects. For example, pemigatinib, an FGFR inhibitor indicated for patients with advanced cholangiocarcinoma harboring *FGFR2* fusions or rearrangements, is associated with hyperphosphatemia as a major side effect, which significantly compromises patient tolerance and long‐term medication adherence. Therefore, the pleiotropic effects and safety profiles of FGF/FGFR signaling must be comprehensively evaluated during therapeutic intervention.

## 6 Conclusion and Perspectives

### 6.1 Challenges in Targeting the FGF/FGFR Signaling

The FGF/FGFR signaling functions as a highly conserved, context‐dependent regulatory network with profound pleiotropy. Dysregulation of FGF/FGFR signaling, whether resulting from pathological hyperactivation or loss‐of‐function, is implicated in multiple diseases such as skeletal dysplasia, metabolic disorders, cancers, and neurological disorders. Despite the deepening understanding of the FGF/FGFR signaling, targeting the FGF/FGFR signaling still faces several challenges.

#### 6.1.1 Structural Biology and Drug Design

In recent years, continuous breakthroughs in structural biology, drug design, and related fields have presented both profound opportunities for innovation and significant challenges in FGF/FGFR signaling pathway research [480]. With the elucidation of key structures such as the FGF23–FGFR–Klotho–HS quaternary complex [29], future efforts can leverage structural biological insights integrated with artificial intelligence (AI)‐driven virtual screening and molecular design to develop next‐generation FGFR inhibitors or FGF analogs with improved subtype selectivity and broader coverage of resistance mutations. Acquired resistance mutations, particularly those affecting gatekeeper residues (e.g., V565 in FGFR2, V550 in FGFR4) and molecular brake residues (e.g., N550), represent core barriers that limit the durability of therapeutic efficacy [511]. There is a pressing need for conformationally selective inhibitors targeting specific subtypes (e.g., FGFR2, FGFR4) or those specifically designed to overcome acquired resistance mutations (e.g., *FGFR2* V565F, *FGFR4* V550L). Furthermore, structure‐based engineering of FGF analogs (e.g., attenuating mitogenic activity while preserving metabolic functions) remains a critical strategy to address the safety bottleneck associated with endocrine FGF analogs.

Accordingly, the synergistic integration of high‐resolution structural biology and AI represents a pivotal strategy to address the above bottlenecks. For instance, high‐resolution x‐ray crystal structures of FGFR have established a structural framework for rational inhibitor design targeting distinct functional states. Meanwhile, AI‐driven protein structure prediction (e.g., AlphaFold2, RoseTTAFold) can perform virtual screening with demonstrated efficacy for kinase targets. Collectively, the integration of these cutting‐edge approaches will facilitate the development of next‐generation FGFR inhibitors or FGF analogs with robust activity and enhanced subtype selectivity.

#### 6.1.2 Metabolic Regulation

Furthermore, building on the “threshold model” and “spatiotemporal specificity” principles established in FGF‐mediated metabolic regulation, recent work has proposed a novel concept of dose‐dependent critical points in FGF efficacy, offering important insights into the precise delivery, dosage optimization, and temporal control of future therapeutics [49]. Beyond endocrine FGF family‐dominated paradigm of metabolic regulation, recent studies have revealed that paracrine FGF4 and FGF1 also participate in metabolic control: Upon binding to FGFR1c, they activate the Ca^2+^/AMPK signaling axis, thereby directly promoting glucose uptake in skeletal muscle and adipose tissue to lower blood glucose levels [512]. This finding challenges the conventional view that endocrine FGFs serve as the primary mediator of metabolic function, thus providing a new perspective for the treatment of diabetes and metabolic disorders [154, 512]. Critically, the FGF/FGFR signaling system does not operate in isolation; instead, it is embedded within an intricate regulatory network involving multiple important signaling pathways, including EGFR signaling and gut microbiota metabolism. This crosstalk sustains tissue homeostasis under physiological conditions, whereas in pathological contexts, it contributes to disease initiation, progression, and drug resistance. Therefore, comprehensive mapping of these contextual interactions is indispensable for developing rational, mechanism‐informed combination strategies and predictive biomarker signatures for FGF/FGFR‐targeted therapeutics.

#### 6.1.3 Gut Microbiota

In recent years, accumulating evidence has revealed the bidirectional regulatory effects between FGF/FGFR signaling and the gut microbiota along its metabolites, showing its integral role in systemic metabolic regulation, barrier integrity, and immune homeostasis. A representative example is the BA‐FXR‐FGF19 enterohepatic axis: The gut microbiota converts hepatocyte‐derived primary BAs into secondary BAs, which then activate FXR signaling in ileal epithelial cells to induce the expression of *FGF19* [513]. Upon portal venous transport to the liver, FGF19 binds to FGFR4/β‐Klotho complex and represses the transcription of *CYP7A1*, the rate‐limiting enzyme in BA synthesis, thereby maintaining BA homeostasis [514]. Beyond BAs, short‐chain fatty acids (SCFAs), the principal products of dietary fiber fermentation by the gut microbiota (e.g., acetate, propionate, and butyrate), transduce signals through G protein‐coupled receptors (GPR)‐41 and GPR43 [515]. Notably, butyrate can indirectly modulate the transcriptional levels of FGF ligands or receptors by inhibiting histone deacetylases (HDACs). Moreover, butyrate reinforces intestinal barrier integrity and modulates local immune responses, and butyrate furnishes a favorable microenvironment for FGF‐mediated tissue repair [516]. Under dysbiotic conditions, aberrant expression of FGF19/15 can ensue, contributing to the pathogenesis of metabolic disorders such as obesity, Type 2 diabetes, and metabolic dysfunction‐associated steatotic liver disease (MASLD) [517, 518]. Collectively, these findings underscore a complex and intimate interplay between FGF/FGFR signaling and the gut microbiota, which constitutes a sophisticated bidirectional feedback network.

Nevertheless, our understanding of this network remains nascent. For instance, the precise molecular mechanisms by which SCFAs regulate FGF/FGFR signaling are still poorly defined, and the tissue‐specific differential regulation of FGF/FGFR signaling by microbial metabolites awaits further elucidation. Moreover, the gut microbiota exhibits substantial inter‐individual variability, which poses considerable challenges for precise interventions targeting the microbiota–FGF/FGFR signaling axis. Given that different individuals can respond markedly differently to the same intervention, developing individualized regulatory strategies targeting FGF/FGFR signaling and gut microbiota will be a critical direction for future research. The above limitations suggest that a multi‐omics integration strategy is required to deeply dissect the interaction between gut microbiota and FGF signaling. Combining metagenomics, metabolomics, and transcriptomics, along with longitudinal sampling analysis, can help distinguish causal mechanisms underlying correlations. These strategies are expected to provide feasible technical routes for precision interventions targeting the gut microbiota‐FGF signaling axis.

#### 6.1.4 Liquid–Liquid Phase Separation

In recent years, LLPS has emerged as a fundamental biophysical mechanism governing spatiotemporal regulation of FGF signaling across multiple subcellular and physiological compartments. At the plasma membrane, FGF2 can undergo LLPS with HS to form condensates that augment FGFR interaction and ERK activation, ultimately regulating cell proliferation and differentiation [519]. Notably, high concentrations of HS can inhibit this process, suggesting that HS influences the intensity of FGF signaling by regulating LLPS efficiency. Furthermore, within BMSCs, nuclear FGF2 employs LLPS to control rRNA transcription and cell proliferation, a regulatory axis that further shapes rDNA chromatin architecture through cooperative interaction with STAT5 [520]. In addition, FGFBP1 itself is also directly involved in the LLPS process. In acute liver injury, FGF6 secreted by skeletal muscle affects the efficiency of its release from the cell membrane by regulating the LLPS dynamics of FGFBP1; the released FGFBP1 forms co‐condensates with FGF5 in the liver through LLPS, activates hepatocyte proliferation signals, and drives liver regeneration [46]. Collectively, the aforementioned findings indicate that LLPS is the core mechanism by which FGF signaling exerts its functions at the extracellular, nuclear, and inter‐organ levels.

### 6.2 Limitations of Current Therapeutic Strategies

Despite substantial advances in therapeutic strategies targeting the FGF/FGFR signaling, several key limitations of current approaches impede their clinical translation and widespread application. This section provides a systematic, mechanism‐informed analysis of these limitations of current therapeutic strategies from three perspectives: drug delivery and tissue selectivity, FGFR‐targeted CAR‐T‐cell therapy, and clinical detection with stratified treatment.

#### 6.2.1 Drug Delivery and Tissue Selectivity

The clinical translation of FGF/FGFR‐targeted biologics and small‐molecule therapeutics is significantly constrained by pharmacokinetic and biodistribution limitations. The short half‐life of recombinant FGF proteins (necessitating frequent administration) and the poor tissue distribution selectivity of small‐molecule inhibitors collectively limit therapeutic efficacy [521, 522]. The therapeutic outcome of FGF/FGFR signaling intervention is highly dependent on achieving adequate drug concentrations and sustained exposure in target tissues. For indications requiring FGF/FGFR signaling activation, recombinant FGF proteins are rapidly degraded by proteases following local application, resulting in short‐lived effects and necessitating frequent dosing, whereas systemic administration carries the risk of off‐target effects. For indications requiring FGF/FGFR signaling inhibition, although small‐molecule inhibitors are amenable to oral or intravenous delivery, they often fail to adequately penetrate the blood–brain barrier to reach central nervous system tumors or achieve sufficient selective accumulation in tumor tissues.

To address these pharmacokinetic and biodistribution barriers, future research should prioritize the development of targeted delivery systems based on nanoparticles, hydrogels, exosomes, or in situ sustained‐release technologies. For instance, local delivery of FGF proteins for tissue repairs from biomaterial encapsulation extends their duration of action, whereas in tumor therapy, active targeting strategies, such as ADCs directed against tumor‐associated FGFR isoforms or BBB‐penetrating nanocarriers, may be employed to deliver FGFR inhibitors selectively to tumor tissues or across the blood–brain barrier. Critically, spatiotemporally precise regulation holds promise for enhancing therapeutic efficacy while substantially reducing off‐target toxicity.

#### 6.2.2 FGFR‐Targeted CAR‐T‐Cell Therapy

The integration of FGFR‐targeting strategies and CAR‐T‐cell therapy represents a highly promising direction in tumor immunotherapy. Unlike conventional FGFR small‐molecule inhibitors, FGFR‐targeted CAR‐T cells function as living, self‐replicating therapeutics, enabling high‐affinity, MHC‐independent binding to FGFR proteins on tumor cell surfaces and triggering potent, serial target cell lysis [452]. To date, the development of FGFR‐targeted CAR‐T‐cell therapy has been almost exclusively focused on the FGFR4, with its primary indication being pediatric RMS [452]. In 2025, the US National Cancer Institute (NCI) launched the world's first Phase I clinical trial of FGFR4‐targeted CAR‐T‐cell therapy (NCT06865664). However, FGFR4‐monospecific CAR‐T cells display suboptimal efficacy against aggressive tumor subclones with low FGFR4 expression. To address this limitation, dual‐targeting CAR‐T has emerged as a leading optimization strategy [357]. By co‐expressing two independent CARs targeting FGFR4 and CD276 on the same T cell, this approach effectively circumvents the challenge of heterogeneous tumor antigen expression [357].

Despite its conceptual promise, the clinical application of FGFR‐CAR‐T‐cell therapy in solid tumors, particularly RMS, still faces significant hurdles, including intratumoral antigen heterogeneity (a challenge shared with small‐molecule inhibitors) and T‐cell exhaustion. Beyond RMS, the integration of FGFR targeting with CAR‐T‐cell technology holds potential for expansion to other FGFR‐driven solid malignancies, such as HCC and CRC. To overcome T‐cell dysfunction, rational combination strategies with ICIs or cytokines designed to rejuvenate exhausted T cells may further enhance efficacy.

#### 6.2.3 Clinical Detection and Stratified Treatment

Accurate identification of patients whose diseases are molecularly driven by aberrant FGF/FGFR signaling is a prerequisite for realizing the therapeutic potential of targeted interventions. This necessitates robust, clinically actionable biomarker frameworks that integrate genomic, transcriptomic, and functional readouts to distinguish true oncogenic dependency from passenger alterations. To date, no standardized clinical assay exists to reliably quantify FGF/FGFR signaling dependence in routine practice. Future efforts should focus on establishing unified protocols for the detection of *FGFR1–4* gene mutations, amplifications, fusions, and expression profiles, while concurrently developing dynamic monitoring technologies based on liquid biopsies (e.g., circulating tumor DNA and exosomal RNA). Moreover, exploring non‐FGFR biomarkers, such as Klotho expression levels, FGFBP concentrations, or downstream signaling pathway activity scores, will enable multidimensional identification of populations likely to benefit, thereby maximizing therapeutic index and minimizing off‐target toxicity.

### 6.3 Future Perspectives

The FGF/FGFR signaling system remains a high‐priority frontier in both basic and clinical research. Looking ahead, addressing the multifaceted challenges above will not come from any single approach; it will require integrating multiple complementary strategies. First, structure‐guided drug design, combined with AI‐driven lead compound discovery, will accelerate the development of next‐generation inhibitors with better selectivity and broader coverage against resistance mutations. Second, nanotechnology‐mediated targeted delivery systems, including nanoparticles, hydrogels, and exosomes, offer solutions to the short half‐life and poor tissue distribution of current therapeutics. Third, FGFR‐targeted CAR‐T‐cell therapy, though still in its early stages, represents a paradigm shift in immunotherapy for FGFR‐driven malignancies. Fourth, the establishment of standardized, regulatory‐grade biomarker assays for detecting FGF/FGFR signaling, including liquid biopsy‐based dynamic monitoring and panels of non‐FGFR biomarkers, will make true stratified treatment a reality. Finally, mechanism‐based combination regimens that co‐target the FGF/FGFR axis along with complementary pathways such as EGFR, immune checkpoints, or gut microbiota‐derived metabolites hold promise for achieving synergistic effects while keeping resistance in check.

Collectively, it is anticipated that the convergence of these multidisciplinary approaches will enable precise, context‐dependent modulation of FGF/FGFR signaling, ultimately providing safer and more effective therapeutic options for patients with aberrant FGF/FGFR signaling‐related diseases.

## Author Contributions

Huakan Zhao and Yongsheng Li conceived the review and were responsible for funding acquisition. Miaoyu Song, Xi Liu, Yang Xiao, and Huakan Zhao drafted the manuscript and prepared the figures and tables. Yongsheng Li contributed the modification of the manuscript. All authors contributed to the discussion and revision and the final version.

## Ethics Statement

The authors have nothing to report.

## Conflicts of Interest

The authors declare no conflicts of interest.

## Data Availability

The authors have nothing to report.
